# The Olfactory Transcriptomes of Mice

**DOI:** 10.1371/journal.pgen.1004593

**Published:** 2014-09-04

**Authors:** Ximena Ibarra-Soria, Maria O. Levitin, Luis R. Saraiva, Darren W. Logan

**Affiliations:** 1 Wellcome Trust Sanger Institute, Wellcome Trust Genome Campus, Hinxton, Cambridge, United Kingdom; 2 European Molecular Biology Laboratory - European Bioinformatics Institute (EMBL-EBI), Wellcome Trust Genome Campus, Hinxton, Cambridge, United Kingdom; University of Michigan, United States of America

## Abstract

The olfactory (OR) and vomeronasal receptor (VR) repertoires are collectively encoded by 1700 genes and pseudogenes in the mouse genome. Most OR and VR genes were identified by comparative genomic techniques and therefore, in many of those cases, only their protein coding sequences are defined. Some also lack experimental support, due in part to the similarity between them and their monogenic, cell-specific expression in olfactory tissues. Here we use deep RNA sequencing, expression microarray and quantitative RT-PCR in both the vomeronasal organ and whole olfactory mucosa to quantify their full transcriptomes in multiple male and female mice. We find evidence of expression for all VR, and almost all OR genes that are annotated as functional in the reference genome, and use the data to generate over 1100 new, multi-exonic, significantly extended receptor gene annotations. We find that OR and VR genes are neither equally nor randomly expressed, but have reproducible distributions of abundance in both tissues. The olfactory transcriptomes are only minimally different between males and females, suggesting altered gene expression at the periphery is unlikely to underpin the striking sexual dimorphism in olfactory-mediated behavior. Finally, we present evidence that hundreds of novel, putatively protein-coding genes are expressed in these highly specialized olfactory tissues, and carry out a proof-of-principle validation. Taken together, these data provide a comprehensive, quantitative catalog of the genes that mediate olfactory perception and pheromone-evoked behavior at the periphery.

## Author Summary

The sense of smell in mice involves the detection of odors and pheromones by many hundreds of olfactory and vomeronasal receptors. The genes that encode these receptors account for around 5% of the whole gene catalog, but they are poorly understood because they are very similar to each other, and are thought to be turned on randomly in only a small number of cells. Here we use multiple gene expression technologies to curate and measure the activity of all the genes involved in the detection of odors and find evidence of many new ones. We show that most genes encoding olfactory and vomeronasal receptors have complex, multi-exonic structures that generate different isoforms. We find that some receptors are consistently more abundant in the nose than others, which suggests they are not turned on randomly. This may explain why mice are particularly sensitive to some odors, but less attuned to others. We find that overall males and females differ very little in gene expression, despite having altered behavioral responses to the same odors. Thus diversity in receptor expression can explain differences in odor sensitivity, but does not appear to dictate whether sex pheromones are differentially detected by males or females.

## Introduction

Olfaction is used for locating and discriminating between food sources, but also plays a fundamental role in social communication between individuals. Mice heavily rely on their sense of smell to distinguish between animals from their own and different species, and to determine their identity [1]. Additionally, upon detection of specific semiochemical cues, these animals show certain behavioral responses, many of which are stereotypical and have been well characterized [2]–[6]. The mammalian olfactory system is formed by the olfactory mucosa (OM) and the vomeronasal organ (VNO) and is dedicated to sensing odorants and pheromones present in the environment. These cues are detected via olfactory (OR), trace-amine associated (TAAR), vomeronasal (VR) and formyl-peptide (FPR) receptors expressed by the sensory neurons in the epithelia of these organs. The OM detects mainly airborne molecules while the VNO identifies both volatile and non-volatile compounds [7]. The importance of a finely tuned olfactory system is reflected in the amount of genes specialized for the detection of odorants and pheromones. In the most recent assembly of the reference mouse genome (GRCm38) over 1,200 genes are annotated as coding for ORs and around 530 for VRs with a smaller number of TAAR and FPR genes. Together they comprise almost 5% of the complete gene catalog.

A large proportion of OR and VR gene repertoires have been identified through computational methods, based on homology searches to a few experimentally described reference receptors [8]. Accordingly most only have their protein coding sequences annotated in genomes. Indeed, for some of the genes annotated as VRs or ORs there is a complete absence of supporting evidence for them being expressed in the VNO or OM. ORs are also expressed in non-olfactory tissues, including the kidney, heart, lung, and testes [9], [10], where they have been shown to work as chemoreceptors in human sperm chemotaxis [11], [12]. This has raised the question of whether all OR genes encode true olfactory receptors [13]. Most olfactory sensory neurons express only one OR or VR gene, in a monoallelic fashion [14]. Consequently only a small proportion of cells in each epithelium express any given receptor, which makes their detection challenging. Furthermore, high levels of sequence similarity within OR, and particularly VR subfamilies, means it is very difficult to ensure specificity when using hybridization based detection methods [9], [15].

Among the behavioral responses elicited through olfactory signals, many are clearly distinct between adult male and female mice, including sexual conduct [3], [4], [6], aggressive responses to intruders [2], and parental care [16], but the mechanisms that ensure such differentiated responses have not yet been fully elucidated in mammals [17]. In silk moths, this is achieved by only males expressing the receptor *BmOR-1* in their antenna, which detects the female-specific sex pheromone bombykol [18]. In contrast, the *Drosophila* sex pheromone cVA is detected by both sexes and elicits dimorphic behavior by routing the signal via different third order neuronal circuits deep in the brains of males and females [19]. Sexual dimorphism in pheromone receptor expression has been reported in rats [20], but the best defined mammalian example is the detection of the male-specific pheromone ESP1 by Vmn2r116, which is capable of eliciting lordosis behavior specifically in female mice. Mice of both sexes appear capable of detecting this pheromone, suggesting the differential response is due to modifications in the downstream neural circuitry [4].

To determine the full receptor repertoire expressed in the mouse VNO and OM, and assess whether sexual dimorphism in olfactory-mediated behavior can be explained by differential gene expression in these organs, we used RNAseq to profile their transcriptomes in male and female mice. We show that a very high proportion of the annotated receptors are indeed expressed in the olfactory system, we experimentally characterize their full length transcripts for the first time and compare their abundances to previous estimates. There are a few differences in expression between the two sexes but only very minor distinctions in the levels of the receptor repertoires. However, genome-wide expression analysis revealed a large number of novel genes in olfactory tissues and some inter-individual variation for subsets of genes.

## Results

### Expression profile of mouse VNO and OM

We conducted deep RNA-sequencing in whole VNO and OM of three adult male and three adult female biological replicates. Due to their small size, each VNO replicate was pooled from three genetically identical animals; each OM replicate was from a single animal. The VNO samples, composed of the sensory neuroepithelium, progenitor and non-neuronal supporting cells, underlying glandular and cavernous tissue and a blood vessel with blood [21], [22], yielded a mean of 37.1 million (±3.6 million) short paired-end fragments per sample (Table 1). The whole OM samples, including the main olfactory epithelium and underlying lamina propria, non-neuronal supporting cells, glandular tissue and blood vessels with blood [23], yielded 46.4 million (±4.3 million) fragments on average (Table 1). From these, approximately 84% were mapped unambiguously to the genome. To estimate expression levels, we counted the number of uniquely mapped fragments assigned to each annotated gene. We then normalized to account for the length of the gene and the depth of sequencing to obtain FPKM values (fragments per kilobase of exon sequence per million fragments) [24]. The expression estimates for all genes in each replicate are listed in Datasets S1, S2.

**Table 1:** Number of fragments obtained for each VNO and OM sample.

	VNO						
Sample	total	Mapped		Multireads		Total in genes	
**male1**	33,829,828	30,039,039	88.79%	2,494,594	7.37%	24,822,223	82.63%
**male2**	34,334,069	30,122,293	87.73%	2,448,849	7.13%	24,487,416	81.29%
**male3**	33,452,308	29,099,459	86.99%	2,398,175	7.17%	24,024,221	82.56%
**female1**	38,989,649	33,011,207	84.67%	2,647,147	6.79%	26,624,869	80.65%
**female2**	41,267,287	35,156,127	85.19%	1,931,707	4.68%	28,660,043	81.52%
**female3**	40,783,743	36,104,171	88.53%	3,074,369	7.54%	29,769,226	82.45%
**mean**	37,109,481	32,255,383	87.0%	2,499,140	6.8%	26,398,000	81.9%

	OM						
Sample	total	Mapped		Multireads		Total in genes	
**male1**	47,449,378	45,781,369	96.48%	2,618,334	5.52%	38,380,328	83.83%
**male2**	45,919,675	44,717,520	97.38%	3,072,143	6.69%	37,128,063	83.03%
**male3**	45,436,958	44,149,432	97.17%	6,273,564	13.81%	34,724,193	78.65%
**female1**	41,096,169	39,819,306	96.89%	4,280,273	10.42%	31,970,108	80.29%
**female2**	53,985,044	52,300,921	96.88%	6,779,517	12.56%	41,287,512	78.94%
**female3**	44,548,659	43,497,165	97.64%	5,109,957	11.47%	35,366,481	81.31%
**mean**	46,405,981	45,044,286	97.1%	4,688,965	10.1%	36,476,114	81.0%

The number of paired-end fragments (comprising two 75 nucleotide long reads) is indicated for the three male and three female replicates with the mean below. The number and proportion of fragments that could be mapped to the mouse genome is indicated, along with those that have equally good alignments to two or more locations in the genome, also called multireads. ‘Total in genes’ corresponds to the number and proportion of mapped reads that fall within an annotated exon in the Ensembl database.

**Figure 1:**
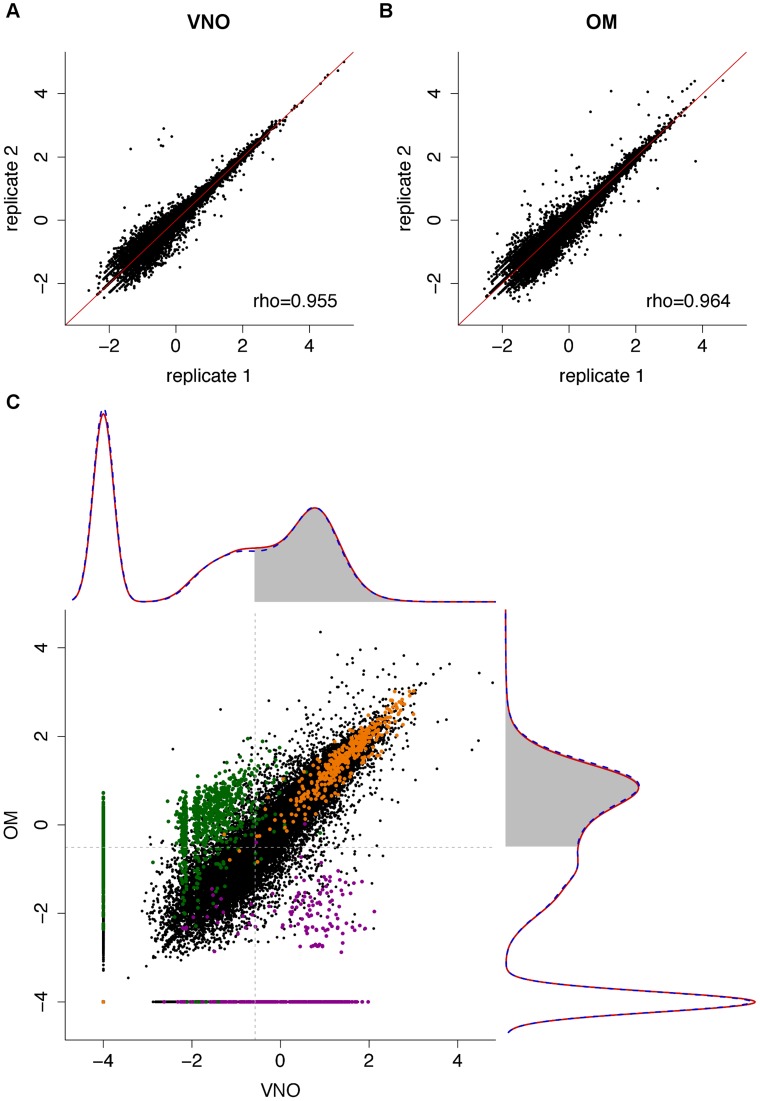
The transcriptomes of the VNO and OM. (A–B) Representative scatter plots of the log_10_ FPKM values for all genes in two biological replicates for the VNO (A) and OM (B). Spearman correlations were computed and the rho values are indicated (see Figure S1 for all pairwise comparisons). (C) The VNO and OM transcriptomes show a bimodal distribution. Density curves were computed for the mean log_10_ FPKM expression values of males (dotted blue) and females (red); 0.0001 FPKM was added to avoid computing log_10_(0). The VNO is shown in the x-axis and the OM in the y-axis. The shaded region indicates genes that have a 0.25 or higher probability of being in the highly-expressed distribution. The scatter plot in the center depicts the expression levels of all genes in the VNO versus the OM. House-keeping genes (orange) tend to be expressed at high and similar levels in both tissues; OR (green) and VR genes (purple) are specifically expressed in their cognate tissue.

We first assessed the variation in gene expression among the three biological replicate samples for each sex and tissue; the correlation values were highly significant between them all (Spearman's rank correlation coefficients of at least 0.95, p-value<2.2×10^−16^; Figure S1). Only small sets of genes are unusually variable among replicates (Figure 1A–B) and the distribution of gene expression is very similar between males and females (density plots in Figure 1C). We therefore averaged the FPKM values for each gene in each sex and tissue. In both tissues a few genes are extremely highly expressed. For example, in the VNO the 14 most abundant genes account for almost 50% of the fragments obtained from the whole tissue. The highest, *Lcn13*, has an average expression of around 97,300 FPKM, but more than 85% of the genes have values below 10 FPKM. A similar distribution is observed in the OM, though less extreme. The most abundant gene, *Bpifb9b*, is expressed at about 22,300 FPKM and the top 261 genes account for half the total expression; again, the overall majority of genes (83.9%) are expressed below 10 FPKM.

A total of 10,552 (28.35%) and 9,881 (26.54%) genes in the VNO and OM respectively have no fragments mapped in any replicate suggesting they are not expressed in that tissue. The expression of the remaining genes shows a bimodal distribution of low- and high-expressed genes (density plots in Figure 1C), characteristic of RNAseq datasets [25]. These can be decomposed into two normal-like overlapping distributions, and each gene can be assigned to either distribution with a degree of confidence. Low-expressed genes typically do not have active chromatin marks, are enriched in non-functional mRNAs and, unlike the high-expressed genes, lack correlative protein expression data [25]. We therefore focused our analysis of differential gene expression on those genes that have at least a 25% probability of being within the highly-expressed distribution: 17,698 genes in the VNO and 17,983 in the OM (see Materials and methods for details). Among the 19,579 genes that are expressed in either tissue, 63.14% (12,363) are differentially expressed with a false discovery rate (FDR) of less than 5%. To explore these further, we selected the genes showing a fold change of four or higher and searched for enriched functional terms. As expected, those expressed higher in the VNO are enriched for VR genes, which are involved in the response to pheromones, odorant-binding and lipocalin-related proteins. Additionally, the calcium signaling pathway, ionic and voltage-gated channel activity, regulation of blood pressure and the immune response are significantly enriched. For the OM, enriched genes are dominated by those encoding ORs and, those involved in the olfactory transduction pathway and sensory perception. In addition, there is enrichment of ionic and ligand-gated channels. In contrast, ‘housekeeping’ genes are expressed at similar levels in both tissues (scatter plot in Figure 1C).

**Figure 2:**
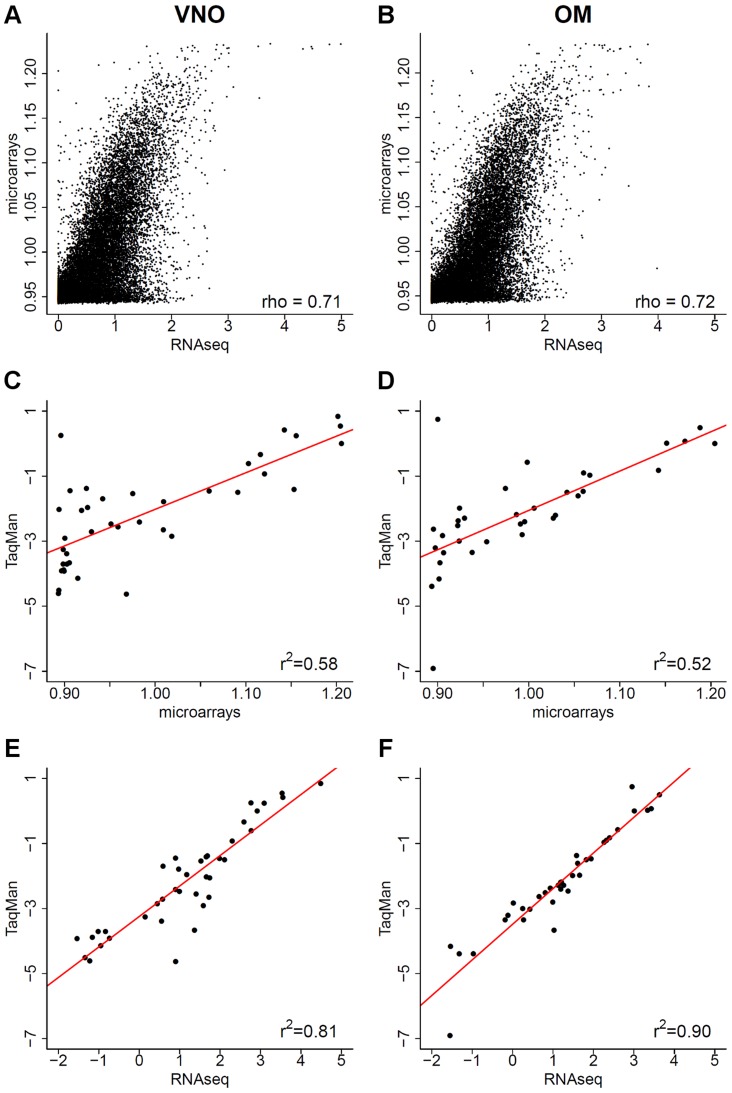
Comparison of RNAseq data to microarrays and qRT-PCR. (A–B) The log_10_ FPKM expression values obtained from RNAseq are plotted against the normalized expression estimates from an Illumina microarray chip for the VNO (A) and OM (B), 1 was added to avoid computing log_10_(0). (C–F) For a subset of genes (Table S1), the log_10_ normalized intensity values obtained from the microarrays (C–D) and from RNAseq in FPKM (E–F) are plotted against the log_10_ RQ values from TaqMan qRT-PCR assays. The Pearson correlation values are indicated and a linear least squares regression is fitted (red).

Another widely used technology to profile gene expression levels is microarrays. For comparison with our RNAseq data we used commercial Illumina expression microarrays to profile six more biological replicate VNO and OM samples. For both tissues, the overall expression values are correlated (Spearman correlation = 0.71 for the VNO, 0.72 for the OM, p-value<2.2×10^−16^). However, the microarray intensity values reach saturation for the highly expressed genes while the RNAseq values keep increasing over two orders of magnitude (Figure 2A–B).

Quantitative real time PCR (qRT-PCR) is accepted as the gold standard for expression profiling, so we next compared both our RNAseq and the microarray expression estimates to a panel of qRT-PCR TaqMan gene expression assays (Figure 2C–F). We included genes with and without a known function in olfactory and vomeronasal signaling that cover the whole range of expression values observed (Table S1). The correlation is considerably higher with the RNAseq data (Pearson correlation r^2^ = 0.81 for the VNO and 0.9 for the OM) than with the microarray values (Pearson correlation r^2^ = 0.58 for the VNO and 0.52 for the OM), indicating that RNAseq is better suited for transcriptome profiling in the olfactory system. Furthermore, the strong correlation between the qRT-PCR and the RNAseq data gives us confidence that these expression estimates are reproducible and specific, and provide a comprehensive characterization of olfactory transcriptomes.

### Sexual dimorphism in olfactory expression profiles

**Figure 3:**
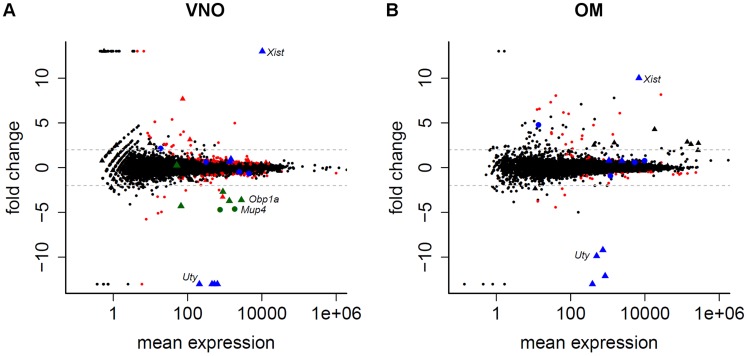
Limited sexual dimorphism in the olfactory system. The mean gene expression is plotted against their log_2_ fold change between male and female samples for the VNO (A) and OM (B). Genes with ±infinite fold changes were assigned to ±13 to ease visualization. Triangles depict genes located on the sex chromosomes. Genes significantly differentially expressed in one tissue (FDR 5%) are red while the 11 genes that are significantly differentially expressed in both tissues are blue. The genes in the VNO plotted in green are eight lipocalins that are highly variable between replicates. Dotted lines indicate a log_2_ fold change of ±2.

To investigate whether sex-specific responses to olfactory cues can be accounted for by transcriptional differences in the VNO and OM, we searched for sexually dimorphic gene expression patterns. We found that the overall transcriptomes are very similar between males and females. In the VNO 282 genes (1.59% of all expressed) are differentially expressed by sex at a 5% FDR. In the OM, only 81 genes (0.45%) reach statistical significance (Figure 3). Furthermore, just 51 and 34 respectively show log_2_-fold changes greater than 2, while the remaining show very slight deviations towards one sex. Only 11 genes are sexually dimorphic in both olfactory tissues. Among these are genes expected to be differentially expressed by sex, such as the X-inactive specific transcript, *Xist*, and four genes on the Y chromosome (*Kdm5d*, *Ddx3y*, *Eif2s3y*, *Uty*). The differential expression analysis between males and females for all genes in each tissue is provided in Dataset S3.

We noted that 110 (39.0%) and 45 (45.5%) of the genes identified as sexually dimorphic in the VNO and OM respectively show unusually high variance (at least a three-fold difference) between any two replicates of the same sex, a likely contributing factor to the dimorphism. Moreover, some subsets of these genes had very similar patterns of variation. For example, a group of eight lipocalins (six of which are significantly dimorphic) all showed at least a 130 fold increase in abundance in one male VNO sample over the two other replicates (Figure 3A, S2A). All other lipocalins do not (Figure S2B), suggesting this variation is unlikely to be a consequence of sample contamination. Due to the small size of the organ, the VNO samples we sequenced were pooled from three mice. Therefore we next extracted RNA from the VNO of 15 individual group-housed males and assessed the expression of four of the most variable lipocalin genes by TaqMan qRT-PCR. Two thirds of the animals showed equivalent expression values but the remaining five had increased expression levels up to ten fold higher than the mean expression across all animals (Figure S2C). Consistent with the RNAseq data, the expression dynamics across each individual animal was the same for the four lipocalins.

### The receptor repertoires

The monogenic expression of receptors in olfactory sensory neurons means each individual receptor is expressed in only a small subset of cells. Therefore the expression of any given receptor within the whole epithelium is low and this has hindered their study in a comprehensive manner. The GRCm38 mouse assembly contains 1,249 annotated OR genes and 530 VR genes. To ensure that these represent the complete repertoires, we took the cDNA sequences for the mouse VR genes as previously reported [26], [27], and aligned them to the genome with BLAST. We recovered 35 Ensembl genes that were not annotated as a VR gene, but that perfectly matched a VR cDNA sequence. These were included in subsequent analyses (Table S2). A similar procedure performed with all OR genes annotated in Ensembl provided four additional genes that have high identity alignments but that had not been annotated.

**Figure 4:**
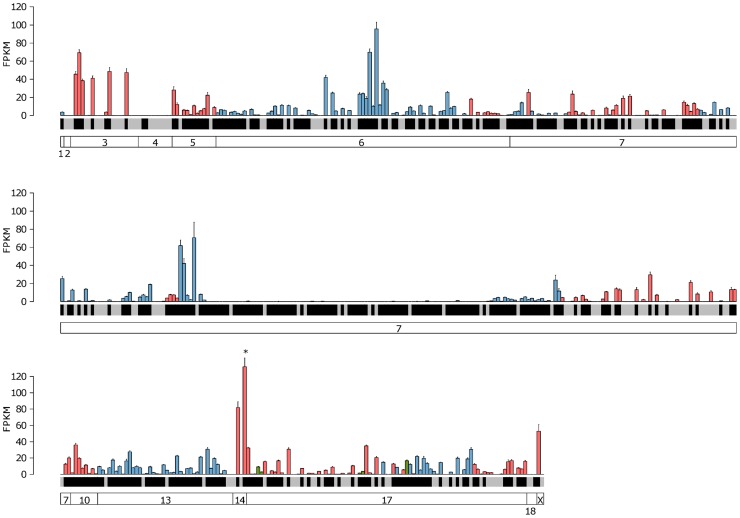
Expression of the complete VR repertoire in the VNO. The mean FPKM expression values are shown for all the VR and formyl peptide receptor (FPR) genes in the VNO. Genes are ordered by their chromosomal location and chromosomes are annotated in the boxes at the bottom. V1R genes are blue, V2R genes are red and FPR genes are green. Black shading below each bar indicates the gene is annotated as a functional receptor, and grey indicates an annotated pseudogene. Error bars represent the standard error of the mean from the six biological replicates. *Vmn2r89* is the highest VR gene expressed and is indicated with an asterisk.

**Figure 5:**
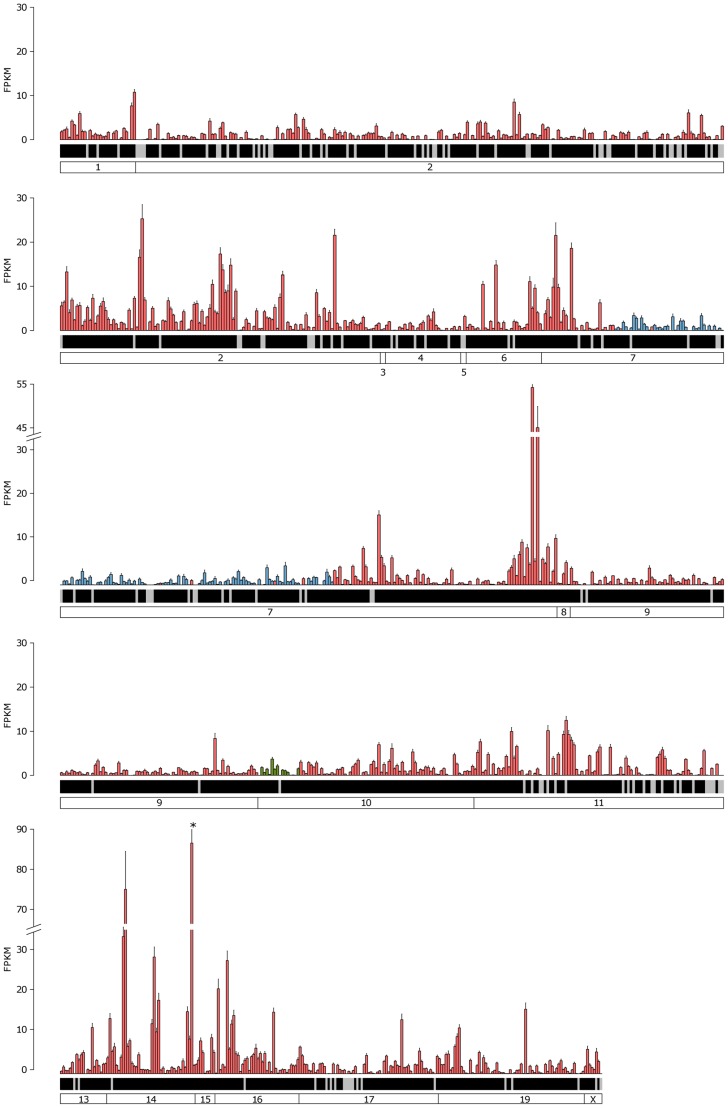
Expression of the complete OR repertoire in the OM. The mean FPKM expression values for all OR and trace amine-associated receptors (TAAR) genes in the OM. Genes are ordered by their chromosomal location and chromosomes are annotated in the boxes at the bottom. Class I OR genes are blue, class II OR genes are red and TAAR genes are green. Black shading below each bar indicates the gene is annotated as a functional receptor, and grey indicates an annotated pseudogene. Error bars represent the standard error of the mean from the six biological replicates. *Olfr1507* is the highest expressed OR gene and is indicated with an asterisk.

We first analyzed the overall expression distribution for each class of receptors in their cognate tissue. In both cases the receptors in the repertoire do not have equal abundances, as may be expected if receptor choice was a random process. Instead we observe a large dynamic range of expression: a few receptors are expressed at high levels and the vast majority of the receptors are expressed at relatively low levels. For the VR genes, the most highly expressed receptor, *Vmn2r89*, has a value of 131.86 FPKM. 42 receptors are expressed above 20 FPKM and the median expression for V2R genes is 0.66 FPKM with 0.45 FPKM for V1R genes (Figure 4, S3A). 416 VR genes (77.6%) have at least one fragment mapped uniquely. From the other 120, 82 are pseudogenes, and 61 have reads that map to several genes (also called multireads). 59 VR genes have no mapped fragments, either unique or multi-mapped, but these are all annotated as pseudogenes. In the case of the OR genes the most abundant, *Olfr1507*, is expressed at 87.54 FPKM, and 11 genes are above 20 FPKM. The median expression is 0.95 FPKM (Figure 5, S3B). Despite their relatively low abundance, 1,180 (94.48%) of all the OR genes have at least one fragment mapped to their exonic region. Of the remaining 69 genes, 50 are annotated as pseudogenes and 16 have multireads that could indicate expression. We found only 9 putatively functional OR genes that have no evidence of expression in the OM whatsoever (*Olfr115*, *Olfr141*, *Olfr504*, *Olfr564*, *Olfr574*, *Olfr834*, *Olfr1053*, *Olfr1061*, *Olfr1367*). Importantly, the expression estimates for both OR and VR genes are consistent between biological replicates suggesting the uneven distribution we observe is stereotypical. We identify no overt pattern in the expression of either VR or OR genes based on cluster or genomic location (Figure 4, 5).

We next asked if any VR genes are expressed in the OM, or if OR genes are found in the VNO, as these may be indicative of specialized olfactory circuits [28]. We found one VR gene, *Vmn2r29*, is expressed in the OM at a level that is higher than the median OR gene expression (1.04 FPKM). This is consistent across all six replicates, suggesting there may be a previously unrecognized mechanism of pheromone detection in the OM (Figure S4). In the case of the VNO, 17 OR genes are expressed higher than the VR gene median (Figure S5), with *Olfr124* as the highest (14.9 FPKM) followed by *Olfr692* and *Olfr1509* (7.4 and 3.1 FPKM respectively). Both *Olfr124* and *Olfr692* consistently display higher FPKM values in the VNO than the OM.

**Figure 6:**
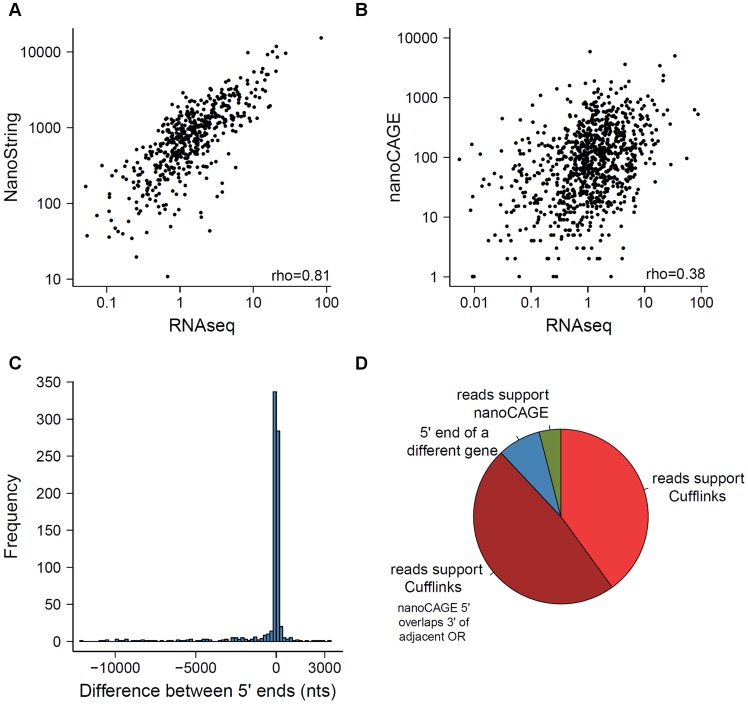
Comparison of methods measuring OR gene expression. A comparison of the expression levels obtained from the RNAseq data to those previously reported using (A) NanoString nCounter and (B) nanoCAGE [23], [29]. The Spearman correlation values are indicated. (C) Comparison of the 5′ of OR transcripts obtained with Cufflinks here, with data estimated by nanoCAGE [29]. The difference in nucleotides between the two ends was calculated; a negative value indicates the 5′ end reported by nanoCAGE is upstream of the one reported by Cufflinks. (D) The receptor genes with most dissimilar 5′ end coordinates can be explained by one of four scenarios; the proportions of each are shown in the pie chart, for the 25 genes with the biggest differences.

In 2012, Plessy *et al.* reported the expression of 955 OR genes using nanoCAGE, a methodology that captures the 5′ end of transcripts and generates short sequence reads around that region [29]. Additionally, Khan *et al.* (2013) profiled 531 OR genes using NanoString nCounter [23]. Both used C57BL/6 sub-strains of mouse, allowing direct gene-level comparison with the data reported here. The NanoString counts are consistent with RNAseq expression estimates (Spearman correlation = 0.81; Figure 6A). The agreement of these two technologies, which are based on very different detection principles, provides support for the accuracy in the quantification of expression of these genes by RNAseq. However, our receptor gene data is only moderately similar to nanoCAGE (Spearman correlation = 0.38; Figure 6B) and the abundance estimates of the OR and VR genes represented on the Illumina microarrays (Spearman correlation = 0.54 for OR and 0.29 for VR genes; Figure S6).

### Annotating receptor genes transcripts in olfactory tissue

**Figure 7:**
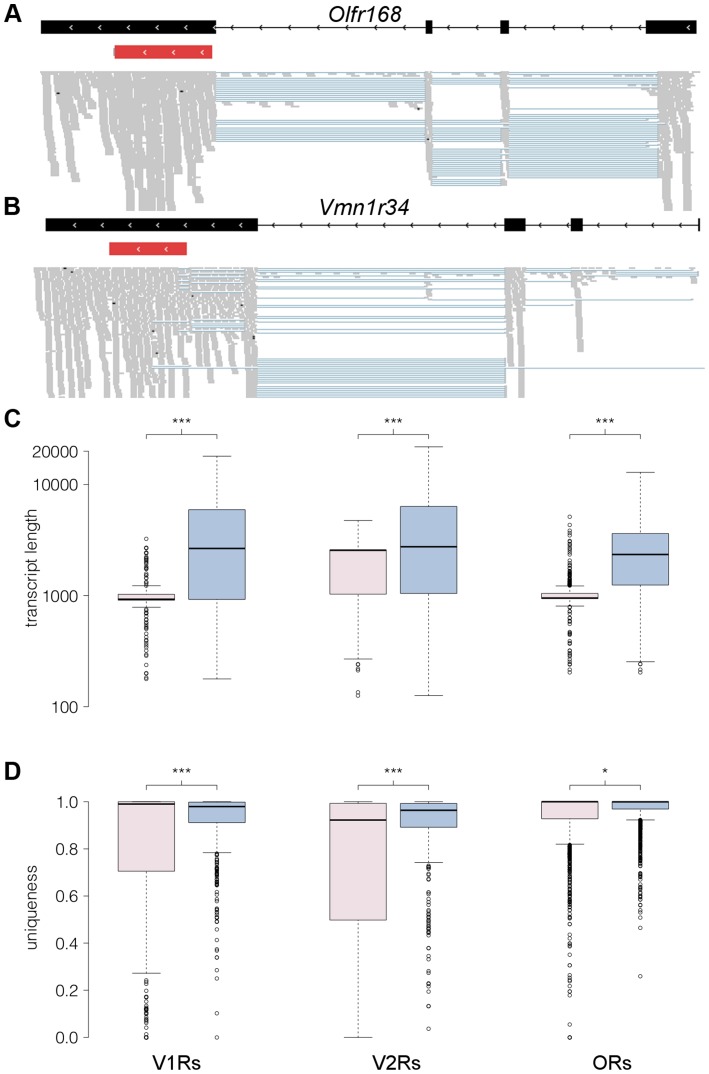
RNAseq provides comprehensive gene models for ORs and VRs. (A–B) An example of new gene models generated for *Olfr168* (A) and *Vmn1r34* (B) are shown in black. Boxes correspond to exons and arrowheads indicate the direction of the gene. The existing Ensembl annotations for the genes are shown in red with their UTRs in grey. New 5′ exons and extended 3′UTRs were identified for both. The mapped RNAseq reads that support the models are below. Each read is drawn in grey and blue lines join read fragments that span exon junctions. Black segments within the reads indicate indels. (C) Boxplots of the transcript length as annotated in Ensembl (pink) or as obtained from the reconstructed models from our RNAseq data (blue) for the V1R, V2R and OR genes. The increase in transcript length is highly significant (*** P<0.0001, two-tailed Mann-Whitney test). (D) As above, but quantifying the proportion of unique sequence for probe design (*** P<0.0001 and *P<0.01, two-tailed Mann-Whitney test). The *uniqueness* corresponds to the proportion of all 100 nucleotide long windows within the transcript that map uniquely to the genome. In all boxplots, outliers are defined as data points that fall outside 1.5 of the inter-quartile range, and are plotted as open circles.

An advantage of RNAseq over the other expression profiling techniques described here is that it is not restricted to a catalog of known transcripts. We therefore used Cufflinks to generate *de novo* assemblies from the sequencing reads in order to identify full length transcripts for OR and VR genes [30]. We sequenced at sufficient depth to produce new, extended receptor gene models for 913 (73.1%) OR and 246 (45.9%) VR genes (the models and their sequences are provided in Datasets S4, S5, S6, S7). We identified additional exons for many of the receptor genes: 866 and 68 OR genes have exons 5′ and 3′ to the coding sequence, respectively; and 163 and 79 VR genes have exons 5′ and 3′ to the coding sequence (Figure 7A–B). OR and V1R genes typically have coding regions that span a single exon, but we identified 54 OR and 15 V1R genes where at least one of the reconstructed transcripts has an intron within the protein coding sequence (as annotated in Ensembl). The predicted open reading frames (ORFs) for most of these transcripts are truncated, due to a premature stop codon. But for 17 OR and 3 V1R genes the ORF is of typical length, and could encode a putatively functional receptor. All but one (*Olfr332*) of these gene models are reported in Ensembl and classified as protein coding (Dataset S8).

We investigated cases of alternative splicing by retaining all the multi-exonic receptor gene models and counted the number of alternative isoforms produced. 70% of VR genes have between 1 and 4 isoforms while 85% of OR genes have 1 to 3 isoforms (Figure S7A). A few receptor genes have more than 8 different isoforms (38 VRs and 10 ORs), however in most of these cases this is due to the presence of several transcription start sites (TSS) or exons that differ in length by just a few nucleotides, so several of the final transcripts differ only very slightly (Figure S7B).

We next calculated the length for each receptor gene based on the existing Ensembl and our new reconstructed models. The median length for both the Ensembl OR and V1R gene models is about 950 nucleotides, while the corresponding reconstructed gene models are now around 2,500 nt long. The median length of Ensembl V2R genes is 2,559 nt, while for the V2R reconstructed gene models it is 2,912 nt (Figure 7C). The lack of experimentally validated UTRs has been a major hindrance for the design of hybridization probes to discriminate between highly similar OR and particularly VR transcripts [9], [15], [31], [32]. We therefore assessed whether our new gene models will help resolve this by determining the proportion of each gene sequence that is unique in the genome. We find a large increase in the proportion of unique sequence in our new extended V1R (P<0.0001, Mann Whitney test) and V2R gene models (P<0.0001, Mann Whitney test); a more modest increase is apparent in OR genes (P = 0.044, Mann Whitney test; Figure 7D).

We next compared the 5′ ends of the OR gene models reconstructed here using Cufflinks, to the proposed transcription start sites (TSS) reported by Plessy *et al.* (2012) using nanoCAGE [29]. A third of the ORs differ in less than 20 nucleotides, and almost 85% are within a 500 nucleotide window (Figure 6C). However 34 OR genes have a discrepancy of more than 5 kb, where the 5′ end proposed by nanoCAGE is upstream of the one found by Cufflinks. We closely examined the sequencing data for the 25 genes with the biggest 5′ differences (Figure 6D). For 24 genes, we were unable to find any sequencing fragments consistent with the TSS proposed by nanoCAGE. In 12 of these cases, the nanoCAGE TSS overlaps with the 3′ UTR of an adjacent OR gene and 2 actually represent the TSS of a different gene (Figure 6D). Only one TSS is correctly inferred by nanoCAGE, where Cufflinks failed to reconstruct the full-length model. Examples of these different scenarios can be found in Dataset S9. Clowney *et al.* (2011) also defined the 5′ end of OR genes using tiling microarrays [33]. We similarly compared the 5′ ends in our reconstructed models to these data and found that a third of the receptor genes differ in less than 100 nucleotides and 80% of the data is contained within a 1.5 kb window (Figure S8).

### Olfactory tissue is a source of novel genes

We extended the analysis of the *de novo* assembly performed by Cufflinks to the full olfactory transcriptomes. This revealed 5,562 and 6,228 loci that have evidence of transcription in the VNO and OM respectively, that do not overlap any annotated genes in the Ensembl database. 40% of these loci are found in both tissues (Table 2). Many of these are located in close proximity to the start or end of annotated genes and are likely to represent unannotated UTRs. Therefore to search for new genes we first excluded all those predictions that lie within 5 kb of cataloged genes. Of the remaining features, about 75% represent single-exon transcripts, leaving 756 and 847 putatively novel multi-exonic genes expressed in the VNO and OM respectively. The genomic coordinates of these are provided in Datasets S10. We cross-referenced these loci with the Ensembl databases to search for alignments to known protein features or overlaps with computationally predicted transcripts. About 30% of these putative genes have known protein domains and 80% lie within transcripts predicted *in silico*.

**Table 2:** Putative novel genes.

	VNO	OM
**Total identified genes**	5,562	6,228
**Shared between tissues**	2,331	2,519
**Within 5 kb of annotated genes**	2,564	2,889
**Putative novel gene models**	2,998	3,339
**Single exon predictions**	2,242	2,492
**Multi-exon predictions**	756	847
**Aligned to a protein domain**	229	258
**Overlap with a predicted transcript**	625	694

Number of novel genes predicted from the RNAseq data (that do not overlap any annotated gene in Ensembl). Predictions within 5 kb of annotated genes were excluded and the remaining are considered putative novel gene models. Putative genes are considered shared between tissues if 50% or more of the gene length is found in both the VNO and OM. In some cases two predicted genes in one tissue can overlap with a single prediction in the other, leading to a different number shared in each. Dataset S6 contains the coordinates for the multi-exonic novel gene models.

**Figure 8:**
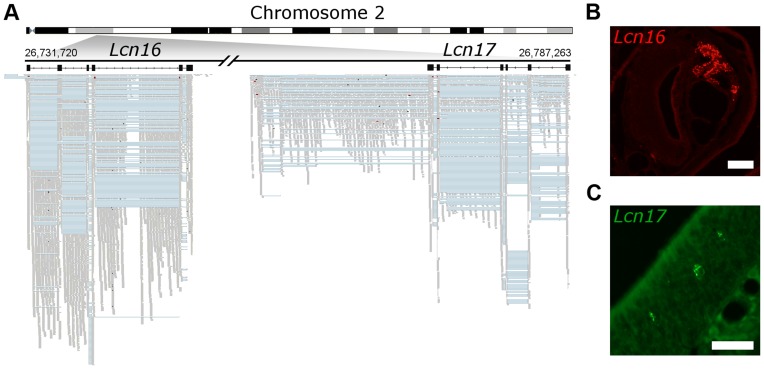
Novel genes are expressed in olfactory organs. (A) Chromosome 2 is schematized on the top and the locus where two previously unidentified genes were found is amplified below. In black are *Lcn16* and *Lcn17* gene models, where boxes correspond to the exons. The mapped RNAseq reads are below: each read is drawn in grey and blue lines join read fragments that span exon junctions. Black segments within the reads indicate indels. (B) *In situ* hybridization reveals *Lcn16* is expressed in glandular tissue of the VNO and (C) *Lcn17* is expressed in cells within the main olfactory epithelium. Scale bars: (B) 100 µm, (C) 50 µm.

Finally, we sought to validate a selection of these putative genes experimentally. We focused on a *de novo* six exon transcript that is extremely highly expressed in the VNO (the 6^th^ most abundant in the transcriptome) and a second, less abundantly expressed novel transcript located adjacent to it in the genome that has OM expression. We cloned full–length transcripts from both these genes and identified ORFs on opposite strands that encode two closely related proteins (Figure 8A). We identified these as novel members of the lipocalin gene family, and named them *Lcn16* and *Lcn17*. A phylogeny of all mouse lipocalins reveals these genes form a distinct sub-clade (Figure S9A), and *in situ* hybridization analyses confirm *Lcn16* is expressed abundantly in glandular tissues of the VNO (Figure 8B, S9B), while *Lcn17* is expressed in a small number of cells in the main olfactory epithelium (Figure 8C, S9C).

## Discussion

In the present study we have reported the transcriptional profile of the two main components of the olfactory system in mice, obtained by RNAseq and expression microarray. By directly comparing the gene expression estimates by these two techniques, using a correlation with TaqMan qRT-PCR as a benchmark, we find RNAseq provides measurements over at least two orders of magnitude greater than the microarray, and thus correlates better with TaqMan values across a large dynamic range.

### Sexual dimorphism

We compared the transcriptomes from VNO and OM of both male and female mice to assess whether differences in gene expression in these tissues could underpin sexually dimorphic behaviors [17]. One mechanism could be to differentially regulate the molecular components involved in cue detection, such as ORs or VRs [20] or in known elements of their signal transduction pathways, under the control of sex-specific hormones. We identified 9 VR genes and 2 OR genes that were significantly more abundant in one gender, but most of these displayed only marginal differences. Only one receptor, *Olfr1347*, had a fold-change in FPKM greater than 2 and none of the other dimorphic transcripts we identified are known to be involved in olfactory or vomeronasal neuron signal transduction. Therefore, we consider it unlikely that the striking dimorphic behavioral responses to some mouse semiochemicals [2], [4], [6], [34] can be solely accounted for by transcriptional differences at the level of detection. It remains to be elucidated whether differences in translation and/or protein modification of receptors or signal transduction machinery underlie sexually dimorphic detection of pheromones. Alternatively, both sexes may detect all mammalian olfactory signals equally, but interpret them differently due to sexually dimorphic central circuits [35].

### Transcriptomic novelty in the olfactory system

A high proportion of the most abundantly expressed genes in our datasets, including *S100a5*, *Obp1a*, *Obp1b*, *Lcn3*, *Lcn4*, *Mup4*, *Mup5*, *Dmbt1*, *Bpifa1* and *5430402E10Rik*, have been previously detected in olfactory tissues using other methods [36]–[40], but a major benefit of RNAseq is that it permits novel gene discovery. Olfactory tissue transcriptomes are likely to be a rich source of novel genes for three reasons. Firstly, they are not widely used in transcription based gene discovery and annotation pipelines. Secondly, olfactory organs tend to be enriched in specialized genes with highly restricted expression patterns. Third, genes involved in pheromone detection are often species-specific and functional orthologues are typically lacking in the human genome, which confound their detection by comparative genomic methods [22].

We found a surprisingly high number of novel transcripts that map some distance away from known genes, and encode consistent multi-exonic gene models. Over 200 of these have protein features, suggesting they are indeed novel genes. As a proof of principle we cloned two, *Lcn16* and *Lcn17*, which encode new members of the lipocalin protein family. Consistent with other lipocalins expressed in the VNO *Lcn16* is extremely abundantly expressed in acinar cells of the vomeronasal gland [37], [39], while *Lcn17* is expressed in cells of unknown function that are scattered throughout the main olfactory epithelium. Orthologous ORFs for *Lcn16* and *Lcn17* are found in the same orientation in the rat genome, but synteny is disrupted around this location in the primate lineage and there are no orthologues present in primates or the human genome.

### Expression of the receptor repertoire

A major goal of our study was to investigate the expression of all the OR and VR genes in parallel. To do so requires a technology that is both sensitive enough to detect highly diluted signals and that is capable of distinguishing between very similar paralogues. In an early attempt to characterize the expression of the receptor repertoire, Young *et al.* screened a cDNA library constructed from the olfactory epithelium, using degenerate olfactory receptor probes, and identified the expression of 419 distinct ORs [41]. This approach, however, suffers from biases in the library construction and screening which hinders the identification of certain classes of receptors. High-density oligonucelotide arrays were designed to target the computationally predicted 3′ UTRs of OR and VR genes and probe the expression of all receptors annotated in an early genome assembly [9], [31]. Expression was confirmed for probes against 817 OR and 266 VR genes. Unfortunately these studies used a different strain of mouse and/or the gene-level expression data is not publically available, thus we were unable to compare those abundance estimates with the data reported here. To address this we used a commercially available expression microarray to estimate abundances. Compared with RNAseq we found that microarrays suffer from high levels of noise, possibly due to non-specific hybridization, and reach saturation with highly expressed genes [42]. We were able to detect expression above threshold for only 39.8% of the 1107 OR genes present in the microarray and for 57.4% of the 197 VR genes represented in the array, consequently there are only moderate correlations between microarray and RNAseq receptor abundance estimates. Surprisingly, we also found that previously published nanoCAGE estimates of OR gene expression correlated poorly with our RNAseq data [29]. This is partly because some 5′ nanoCAGE tags were apportioned to the wrong OR transcript (Figure 6D), but other factors may also contribute to the disparity. The mice used by Plessy *et al.* were younger than used here and their tissue was collected by laser capture microdissection which could result in incomplete sampling of the whole epithelium. Moreover, nanoCAGE tags that mapped to multiple locations were distributed by algorithm, while we took a more conservative approach and did not include multi-mapped reads in abundance estimates.

More recently, NanoString nCounter technology has been used to detect OR expression in the OM [15], [23]. Probes could be designed to only approximately half of the predicted OR gene repertoire with confidence, resulting in expression quantification values for 531 OR genes in whole olfactory mucosa. NanoString nCounter is a hybridization probe based method, thus relative measures of abundance between different OR genes are not necessarily accurate. Nevertheless, we found OR gene expression estimates using this very different technology were consistent with our RNAseq data, lending support to both methods.

We obtained evidence of expression for all putatively functional VR genes and all but 9 potentially functional OR genes by RNAseq. We cannot rule out the possibility that these may be expressed at levels below the threshold of detection in our experiments. Indeed one (*Olfr504*) is present in the NanoString dataset where it was reported to be expressed, albeit at a low level [23]. However, some ORs are known to be ectopically expressed in mice [10], [43], [44], thus it is possible they may have evolved extra-olfactory functions. Alternatively, they could be expressed at a different age [23], or they may be cryptic pseudogenes that have disrupted promoter elements and thus are no longer recognized by the machinery regulating olfactory receptor choice. Supporting this is our observation that approximately one third of both OR and VR genes with interrupted ORFs are not expressed in olfactory tissues, a bias that had been noted previously [41]. Experimental disruption of the ORF of an OR allele does not ablate its expression, instead another OR allele is co-expressed in the same cell [45], [46]. However, this phenomenon clearly occurs less frequently with naturally occurring pseudogenes, which probably reflects a parallel degeneration of their regulatory sequences. By comparing the promoter sequences of expressed with non-expressed OR and VR genes and pseudogenes, it may now be possible to identify key genomic motifs that control receptor choice.

### Receptor transcripts are complex

The generation of RNAseq data for a majority of ORs and VRs enabled us to obtain new, significantly extended gene models. The vast majority of receptor genes contain several exons and it is common to observe differential inclusion of these, diversifying the transcript set produced from each gene. In most cases the putative coding sequences of OR and V1R genes span a single exon and the additional exons contains only UTRs. In these instances the functional consequence of alternative splicing is unclear, as the same receptor protein would be generated from each transcript. In other cases alternative transcripts have introns interrupting the coding sequence, resulting in a truncated ORF that is likely to encode a non-functional receptor. However 1.5% and 1.3% of OR and VR genes, respectively, generate transcripts that can theoretically encode multiple, putatively functional receptor proteins. Further work will be necessary to verify the existence of transcripts, and determine their functional consequences.

Most receptors are annotated from comparative genomic studies [8], which means non-coding UTRs are frequently missing. Identifying UTRs is especially useful for VR and OR genes, since they provide additional sequence that is typically more divergent than the ORF. When we compare the complete gene models we have reconstructed with those currently annotated, both the amount of the receptor transcript sequence, and the proportion that is unique between receptors increases substantially. We anticipate this resource will permit the design of more specific probes for NanoString nCounter, TaqMan qRT-PCR, microarray or *in situ* hybridization and thus increase the utility of these techniques in the olfactory system.

## Materials and methods

### Ethics statement

The use and care of animals used in this study was approved by the Wellcome Trust Sanger Institute Animal Welfare and Ethics Review Board in accordance with UK Home Office regulations, the UK Animals (Scientific Procedures) Act of 1986.

### RNA sequencing

All mice used were C57BL/6J, 8 to 10 weeks old and group housed. The VNO was dissected from nine male and nine female animals and the tissue from three animals was pooled to obtain 5 ug of RNA for each biological replicate. Each OM sample was obtained from a single animal. RNA was extracted using the RNeasy mini kit (Qiagen) with on-column DNAse digestion, using a disposable RNAse free plastic grinder to homogenize the sample. All RNA was subsequently quantified with a spectrophotometer and visualized for quality by RNA integrity analysis. mRNA was prepared for sequencing using the TruSeq RNA sample preparation kit (Illumina) with a selected fragment size of 200–500 bp. The VNO samples were sequenced on the Illumina Genome Analyzer II platform and the OM samples on an Illumina HiSeq 2000; both generated 75 bp paired-end reads.

### RNAseq data processing and analysis

Using STAR 2.3 [47], sequencing reads were aligned to the GRCm38 mouse reference genome, annotation version 68 of the Ensembl mouse genome database (http://jul2012.archive.ensembl.org/info/data/ftp/index.html). The number of fragments aligned to each gene was counted using the HTSeq package with the script *htseq-count*, mode *intersection-nonempty*. Any reads that map to multiple locations in the genome (also called multireads) are not counted towards the expression estimates since they cannot be assigned to any gene unambiguously, but these provide evidence of transcription in at least one of the loci to which they map. To compare the expression values across genes and conditions, raw count data was transformed into fragments per kilobase of exon per million fragments (FPKM) with the formula:
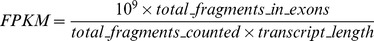



We assessed GC-content biases in our RNAseq data as previously described [48], but observed no correlation between GC-content and fold-change in a differential expression test. Therefore, the FPKM values were not adjusted. Plotting, linear regression and computation of the Pearson's and Spearman correlation was carried out in the R environment (http://www.R-project.org) and sequencing reads were visualized using IGV [49].

### Microarray data generation and analysis

RNA was extracted from the VNO and OM of six C57BL/6J males of 10 weeks of age as previously described. Profiling was performed on the Illumina MouseWG-6 v2.0 Expression BeadChip following the manufacturer's instructions. Variance stabilizing transformation was applied to the data obtained from BeadStudio, which was then quantile normalized using the Bioconductor R package, lumi [50].

### Annotation of the olfactory and vomeronasal receptors in the mouse genome

We are aware that some of the receptor genes are not properly annotated in the Ensembl transcriptome, but are reported as novel genes. To recover the entirety of the VR gene repertoire, we took the cDNA sequences as reported [26], [27] and locally aligned them to the mouse genome with BLAST. Then we identified those alignments that overlap genes not annotated as VRs with 100% identity, and changed their name while preserving the Ensembl identifier. In all cases the coordinates obtained from the alignments were concordant with the annotation. A list detailing the gene names that were changed is reported in Table S2. Furthermore, 19 additional predicted genes have high identity alignments to other VR sequences. Similarly, we aligned with BLAST all the OR cDNA sequences present in Ensembl and recovered four predicted genes that share high similarity to other ORs.

### TaqMan qRT-PCR

To compare the expression estimates form RNAseq and microarrays to those from qRT-PCR, RNA from OM and VNO was extracted, as previously described, from four individual male and four individual female C57BL/6J mice. Predesigned TaqMan gene expression assays were selected to target genes across the full range of expression values obtained by RNAseq (Table S1). They were used on a 7900HT Fast Real-Time PCR System (Life Technologies) according to the manufacturer's instructions. To test for correlation between technologies, mean cycle threshold (Ct) values were obtained from three technical replicates and each normalized to *Actb* and *Eef1a1* expression (chosen because of its similar abundance in both OM and VNO) using the ΔΔCt method. Relative quantity (RQ) values were calculated using the formula RQ = 2^−ΔΔCt^. For validating the inter-individual variation in lipocalin genes, the mean Ct values were obtained from two technical replicates and each normalized to *Actb* using the ΔCt method. RQ values were calculated using the formula RQ = 2^−ΔCt^.

### Fitting distributions for the high- and low-expressed genes

The overall distribution of expression values obtained from RNAseq data is bimodal. It has been proposed that such distribution is the combination of two normal-like distributions of low- and high-expressed genes [25]. Gaussian mixture models can be used to estimate such underlying normal distributions. We used the expectation-maximization algorithm provided in the *Mixtools* Bioconductor package [51], using all genes with at least one fragment count in one replicate, for each tissue. For both transcriptomes, the algorithm converged to optimal values and two distributions were fitted. The algorithm reports, for each gene, its probability of being part of either distribution. Based on this, we arbitrarily considered genes to be expressed if they had a 25% or greater probability of falling in the distribution containing the highly-expressed genes.

### Differential expression analysis

To test for differential expression between sexes and tissues, we used DESeq [52] on the genes defined as expressed. Variation between replicates was calculated with the function *estimateDispersions*, using *per-condition* as the method. Genes were considered to be differentially expressed if they had an adjusted p-value of 0.05 or less (equivalent to a false discovery rate of 5%).

### Functional terms enrichment analysis

The Database for Annotation, Visualization and Integrated Discovery (DAVID) was used to search for enrichment of functional terms and biological processes [53]. In all analyses a background was provided, comprising only the expressed genes used for the relevant analysis. We considered significant those with an adjusted (false discovery rate) p-value smaller than 0.1.

### Discovery of novel genes and transcripts

To search for unannotated genes we performed Reference Annotation Based Transcript (RABT) Assembly [30], using Cufflinks v2.1.1 guided by the Ensembl annotation (version 68). Assembled transcripts from the different replicates were combined with Cuffmerge. In order to extract the candidates with greatest probability of encoding protein coding genes, we cross-referenced all predicted loci to the Ensembl databases using the API [54]. *Ad hoc* perl scripts were used to further refine the gene models produced for VR and OR genes, deleting those predictions that fuse adjacent receptor genes or that are antisense to the annotated gene.

### Validation of novel genes

Full length transcripts of *Lcn16* and *Lcn17* were obtained using 5′ and 3′ RACE kits (Invitrogen) on RNA from VNO and OM tissue following the manufacturer's instructions. All genes with a lipocalin domain (IPR000566) were extracted from Ensembl version 68 and a phylogeny was reconstructed in MEGA using the neighbour-joining method with the Kimura-2 parameter model of substitution [55].

DNA corresponding to the probe-specific regions were synthesized and integrated in pIDT (Integrated DNA Technologies). Templates for *in situ* hybridization probes were amplified by PCR from those plasmids by using the forward primers and reverse primers with or without a T3 promoter site (TATTAACCCTCACTAAAGGGAA) attached to their 5′ end. The primers used were: Lcn16_fw, TGACCATAAGCCTGACCGTG; Lcn16_rv, AGTGCCACATCCACAGAGTG; Lcn17_fw, TTACCCCACTGCCTCCCTTT; Lcn17_rv, TTGTTGGCGTTGGTGCCATA. Digoxigenin (DIG)-labeled sense and anti-sense probes for *Lcn16*, and fluorescein (FLU)-labeled sense and anti-sense probes for *Lcn17*, were synthesized according to the manufacturer's instructions (Roche).

Adult mice (C57BL/6J, 8 weeks old) were anesthetized, and then perfused with 4% paraformaldehyde (PFA). Snouts were dissected out and, post-fixed at 4°C for 2 hours in 4% PFA, then decalcified for another 72 hours by immersion in a 50∶50 mixture of 4% PFA and 0.5 M EDTA. This was followed by immersion in 30% sucrose for 16 hours. The snouts were then embedded in TissueTek O.C.T. (Sakura), and frozen at −20°C. 14 µm cryosections were thaw mounted onto Superfrost Plus slide glasses (Thermo), dried at 55°C for 2 hours, and kept at −20°C until use. Hybridizations were performed overnight at 58°C using standard protocols [56]. Probes were visualized with the direct TSA Kit (FITC or Cy3, Perkin Elmer), or HNPP-Fast Red (Roche), according to the manufacturer's instructions. Slides were mounted with VectaShield (Vector). Sections were observed and photographed with a Leica DM 400B fluorescent microscope, attached to an Olympus DP72 camera.

### Data access

RNAseq data are available in the European Nucleotide Archive (ENA) under accessions PRJEB2572 and PRJEB1365. Microarray data are available in the ArrayExpress database under accession number E-MTAB-2163. The sequences for *Lcn16* and *Lcn17* are available in GenBank under accessions KJ004569 and KJ004570.

## Supporting Information

Figure S1 RNAseq correlations between biological replicates. The three male and three female samples are listed on the diagonal. Above are pairwise comparisons between biological replicates, shown as scatter plots of the log_10_ FPKM expression values for all genes. Below the diagonal the rho value of the Spearman correlation is indicated.
(TIFF)

Figure S2 Expression of a group of lipocalins is highly variable between individuals. Four out of eight lipocalins that are highly variable between biological replicates were validated in an independent set of animals. (A) The expression estimates obtained in the RNAseq data for these four genes. FPKM values are very high for the male2 sample but not for the others. (B) The expression values in the same samples for other lipocalins do not show the same amount of variation. (C) Normalized TaqMan qRT-PCR expression estimates for the variable lipocalins (black) in the VNO of 15 group-housed males. Expression of other three control genes (grey) indicates that the variability observed is specific.
(TIFF)

Figure S3 Expression of receptor genes and pseudogenes differ. Vomeronasal receptor (A) and olfactory receptor genes (B) annotated as functional (black) are expressed at significantly higher levels than those annotated as nonfunctional pseudogenes (grey). (C) When only those annotated as functional are considered, on average V2R genes are more abundant than V1R genes in the VNO, and (D) class II OR genes are more abundant than class I (*** P<0.0001, two-tailed Mann-Whitney test).
(TIF)

Figure S4 Expression of the complete VR repertoire in the OM. The mean FPKM expression values are shown for all the VR and formyl peptide receptors (FPR) genes in the OM; error bars represent the standard error over the mean from the six biological replicates. Genes are ordered by their chromosomal location and chromosomes are annotated in the boxes at the bottom. VIR genes are colored in blue, V2R genes in red and FPR genes in green. Below the plot, the black shading indicates the gene is annotated as a functional receptor, and grey is for pseudogenes.
(TIFF)

Figure S5 Expression of the complete OR repertoire in the VNO. The mean FPKM expression values are shown for all the OR genes and trace amine-associated receptor (TAAR) genes in the VNO; error bars represent the standard error over the mean from the six biological replicates. Genes are ordered by their chromosomal location and chromosomes are annotated in the boxes at the bottom. Class I OR genes are colored in blue, class II OR genes in red and TAAR genes in green. Below the plot, the black shading indicates the gene is annotated as a functional receptor, and grey is for pseudogenes.
(TIFF)

Figure S6 Expression of VR and OR genes in RNAseq compared with microarray expression data. Expression of the VR (A) and OR (B) genes that are present in both the microarray and the RNAseq data. The log_10_ FPKM+1 values from the RNAseq data are plotted in blue, and genes are ordered by decreasing expression level. In red is the log_10_ normalized intensity values from Illumina expression microarrays for the corresponding genes. The dotted black line represents the background intensity level from the microarray. Gene expression values lower than this threshold cannot be distinguished from noise.
(TIFF)

Figure S7 Alternative splicing in VR and OR genes. (A) Histograms representing the number of receptor genes that show different number of multi-exonic isoforms for OR, V1R and V2R genes. (B) Most genes with a high number of isoforms arise from multiple combinations of slight variations in the (a) transcription start site, (b) the differential inclusion of specific exons and (c) the difference in splice sites, which produces exons that differ in length by a few nucleotides. Data shown for *Olfr1420* as an example; in red is the Ensembl gene model, with the UTR regions in grey, and in black the models produced by Cufflinks. Below are the sequencing reads in grey, and blue lines join reads that span exon junctions.
(TIFF)

Figure S8 Comparison of the 5′ end of the OR genes as inferred by Cufflinks or as reported by Clowney *et al.* (2011). The difference in nucleotides between the two ends was calculated; a negative value indicates the 5′ end reported by Clowney *et al.* is upstream of the one reported here, by Cufflinks [33].
(TIFF)

Figure S9 Novel lipocalins are expressed in olfactory tissues. (A) Phylogenetic reconstruction of the novel genes with other members of the mouse lipocalin gene family. *In situ* hybridization with an antisense probe (AS, left) reveals (B) *Lcn16* is expressed in glandular tissue of the VNO, but not within the sensory epithelium (dashed line) and (C) *Lcn17* is expressed within the main olfactory epithelium of the OM (dashed line). No signals are detected using the corresponding sense probes (S, right). Scale bars: (B) 100 µm, (C) 50 µm.
(TIF)

Table S1 TaqMan probes used in qRT-PCR assays. The IDs of the specific assays used are provided.
(DOCX)

Table S2 VR genes are not properly annotated in Ensembl. Ensembl genes that match VR cDNA sequences but are annotated as ‘novel genes’. Those genes that match a sequence with 100% identity were included in our analyses and their name was changed to that of the cDNA sequence they matched (third column).
(DOCX)

Dataset S1 Expression estimates in the VNO. A dataset containing the raw and normalized expression values for all the genes in the VNO, and including the VR and FPR genes subdivided.
(XLSX)

Dataset S2 Expression estimates in the OM. A dataset containing the raw and normalized expression values for all the genes in the OM, and including the OR and Taar genes subdivided.
(XLSX)

Dataset S3 Differential expression analysis between male and female samples. A dataset containing the results of the differential expression analysis between males and females for both the VNO and OM. Genes with an adjusted p-value (padj)<0.05 were considered significant.
(XLSX)

Dataset S4 Extended gene models for the VR repertoire. The reconstructed gene models for VR genes based on the VNO RNAseq dataset, provided in GTF format. The ENSEMBL gene models are also included. Each model has been given a unique gene-transcript_id pair. The Ensembl models are annotated as ENSEMBL_transcript on column 2 and the transcript_id on column 9 corresponds to that of Ensembl. The gene models that differ to the Ensembl ones, are annotated as reconstructed_transcript on column 2. For those exons that overlap with the Ensembl model, the id has been included as a reference (in a field in column 9).
(GTF)

Dataset S5 Sequence of the extended gene models for the VR repertoire. The sequences of the reconstructed gene models for VR genes in FASTA format.
(FASTA)

Dataset S6 Extended gene models for the OR repertoire. The reconstructed gene models for OR genes based on the OM RNAseq dataset, provided in GTF format. The ENSEMBL gene models are also included. Each model has been given a unique gene-transcript_id pair. The Ensembl models are annotated as ENSEMBL_transcript on column 2 and the transcript_id on column 9 corresponds to that of Ensembl. The gene models that differ to the Ensembl ones, are annotated as reconstructed_transcript on column 2. For those exons that overlap with the Ensembl model, the id has been included as a reference (in a field in column 9).
(GTF)

Dataset S7 Sequence of the extended gene models for the OR repertoire. The sequences of the reconstructed gene models for OR genes in FASTA format.
(FASTA)

Dataset S8 List of VR and OR genes that have introns within the annotated coding sequence. The genes and their identifiers are listed, and screenshots from IGV with the visualization of the reads supporting the introns are presented.
(PDF)

Dataset S9 Comparison to the Plessy *et al*. (2012) data. The OR genes with the largest difference in their predicted 5′ end by nanoCAGE or Cufflinks can be explained by four scenarios. These are detailed here and examples are provided.
(PDF)

Dataset S10 Multi-exonic novel gene models. GTF file detailing the multi-exonic novel putative gene models reconstructed for the VNO and OM RNAseq dataset. Column 6 indicates whether the model was predicted from the VNO or OM.
(GTF)
