# Supplementary figures and images for: The Olfactory Transcriptomes of Mice
Source: PLoS Genet. 2014 Sep 4;10(9):e1004593. doi: 10.1371/journal.pgen.1004593 (PMC4154679; doi:10.1371/journal.pgen.1004593)

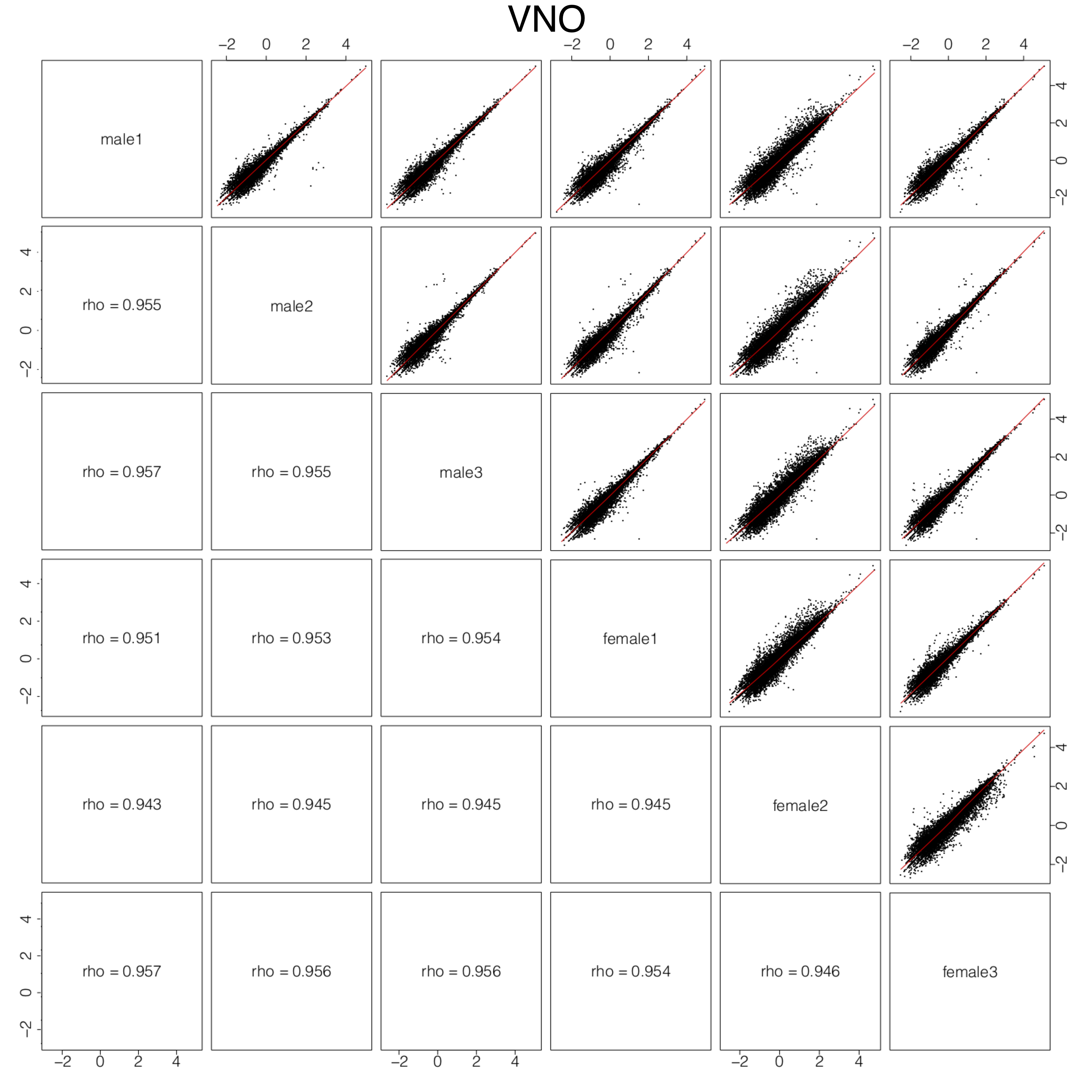

Supplement: Figure S1 — RNAseq correlations between biological replicates. The three male and three female samples are listed on the diagonal. Above are pairwise comparisons between biological replicates, shown as scatter plots of the log10 FPKM expression values for all genes. Below the diagonal the rho value of the Spearman correlation is indicated. (TIFF) [file pgen.1004593.s001.tiff]

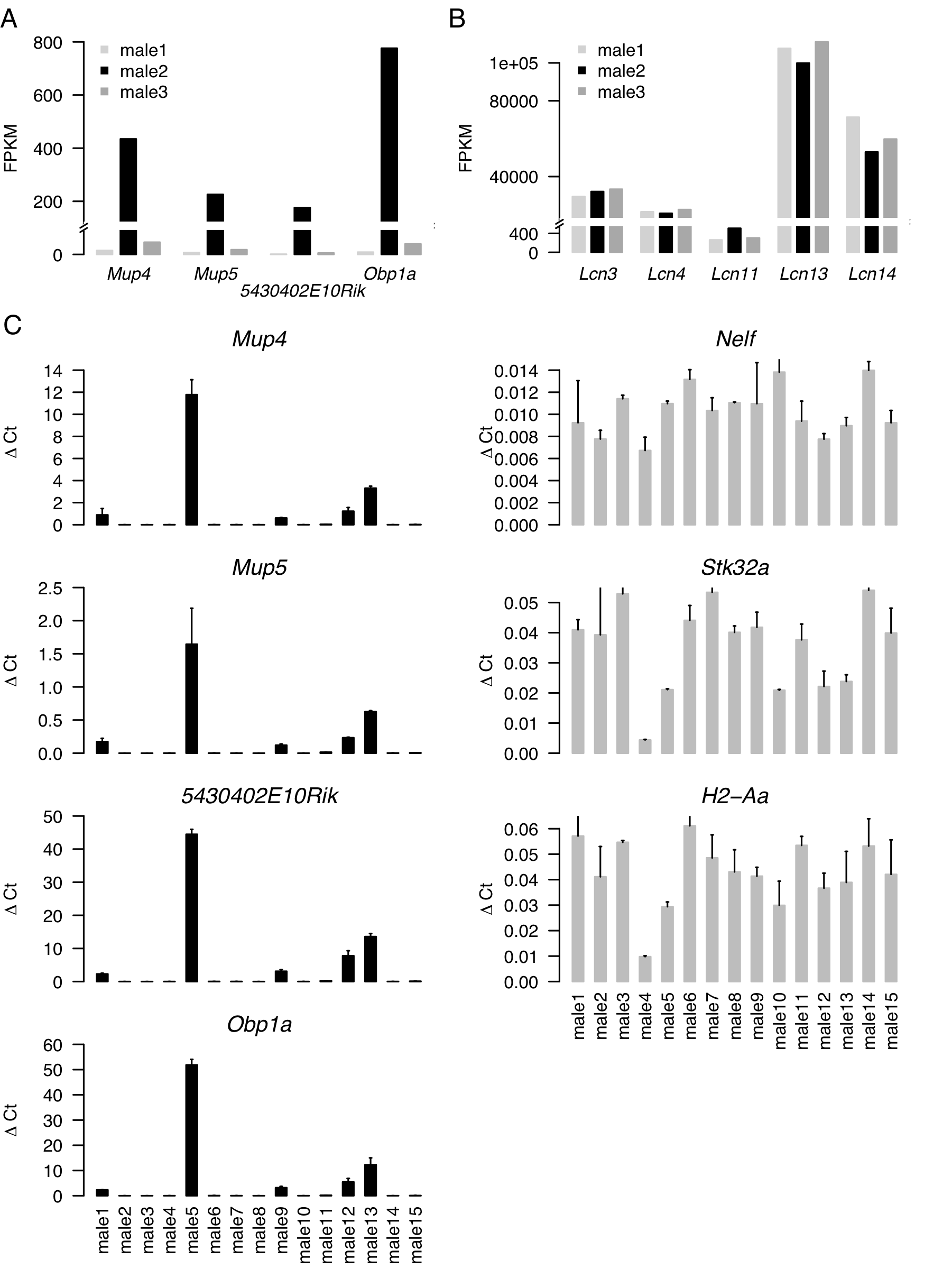

Supplement: Figure S2 — Expression of a group of lipocalins is highly variable between individuals. Four out of eight lipocalins that are highly variable between biological replicates were validated in an independent set of animals. (A) The expression estimates obtained in the RNAseq data for these four genes. FPKM values are very high for the male2 sample but not for the others. (B) The expression values in the same samples for other lipocalins do not show the same amount of variation. (C) Normalized TaqMan qRT-PCR expression estimates for the variable lipocalins (black) in the VNO of 15 group-housed males. Expression of other three control genes (grey) indicates that the variability observed is specific. (TIFF) [file pgen.1004593.s002.tiff]

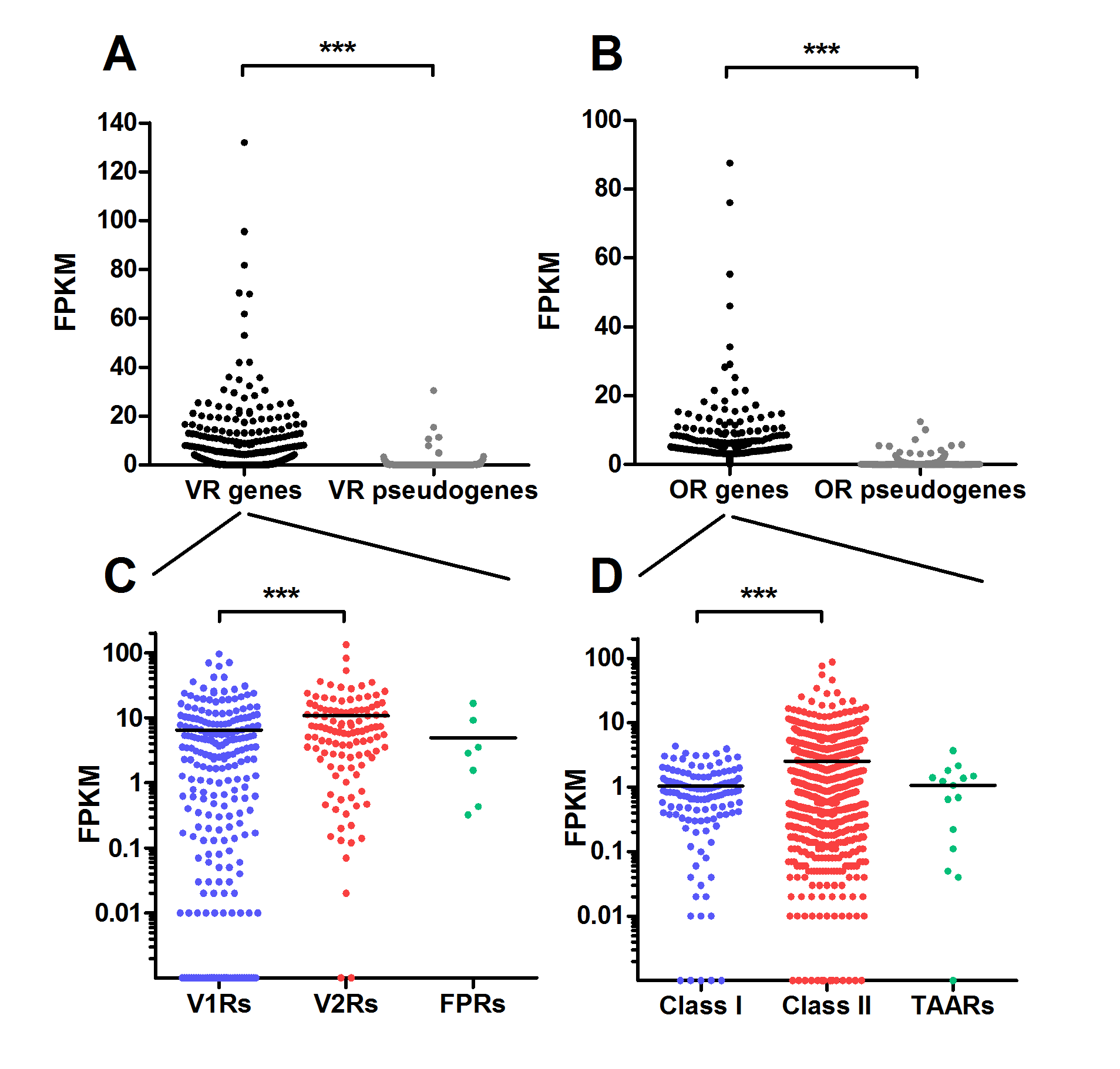

Supplement: Figure S3 — Expression of receptor genes and pseudogenes differ. Vomeronasal receptor (A) and olfactory receptor genes (B) annotated as functional (black) are expressed at significantly higher levels than those annotated as nonfunctional pseudogenes (grey). (C) When only those annotated as functional are considered, on average V2R genes are more abundant than V1R genes in the VNO, and (D) class II OR genes are more abundant than class I (*** P<0.0001, two-tailed Mann-Whitney test). (TIF) [file pgen.1004593.s003.tif]

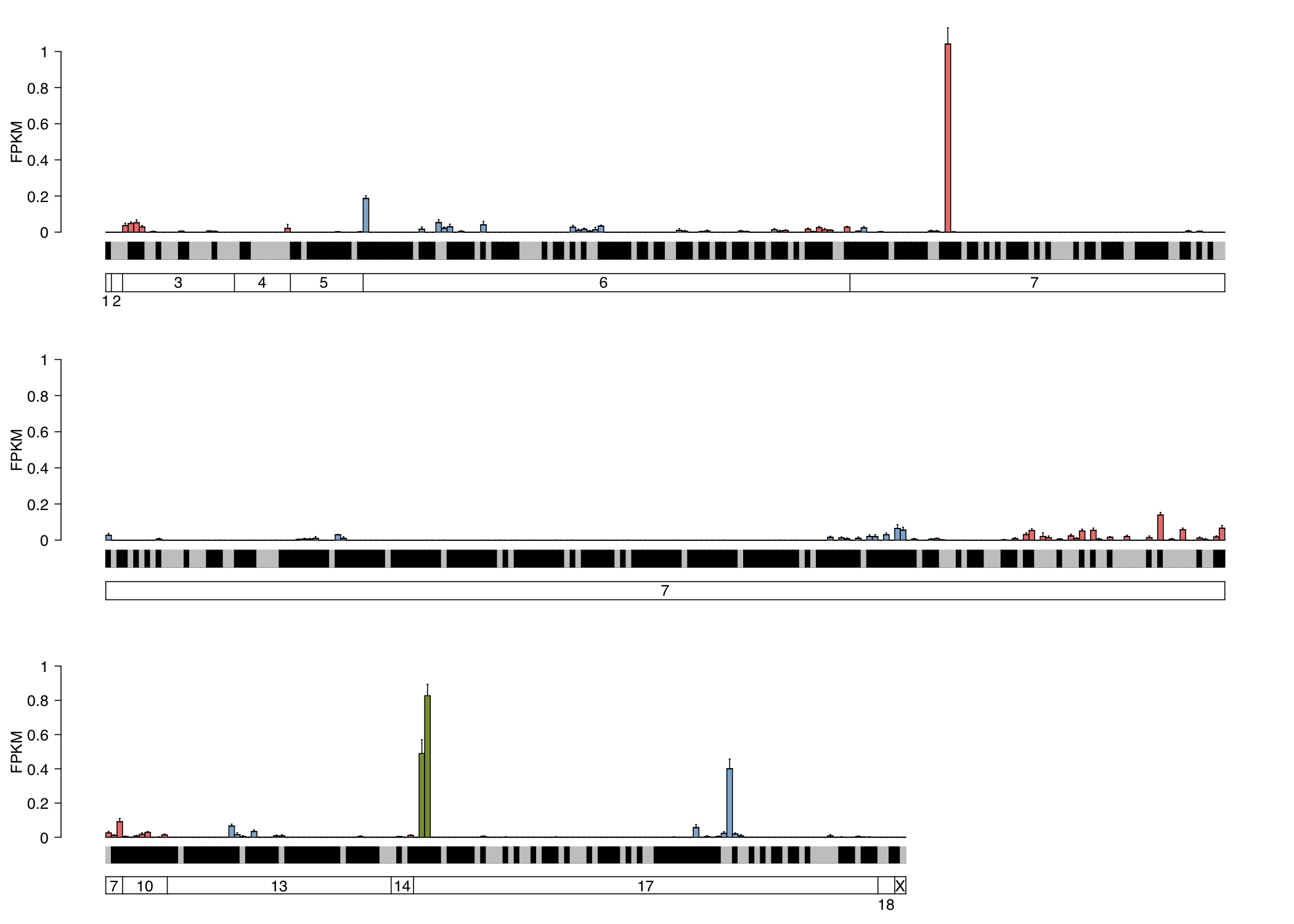

Supplement: Figure S4 — Expression of the complete VR repertoire in the OM. The mean FPKM expression values are shown for all the VR and formyl peptide receptors (FPR) genes in the OM; error bars represent the standard error over the mean from the six biological replicates. Genes are ordered by their chromosomal location and chromosomes are annotated in the boxes at the bottom. VIR genes are colored in blue, V2R genes in red and FPR genes in green. Below the plot, the black shading indicates the gene is annotated as a functional receptor, and grey is for pseudogenes. (TIFF) [file pgen.1004593.s004.tiff]

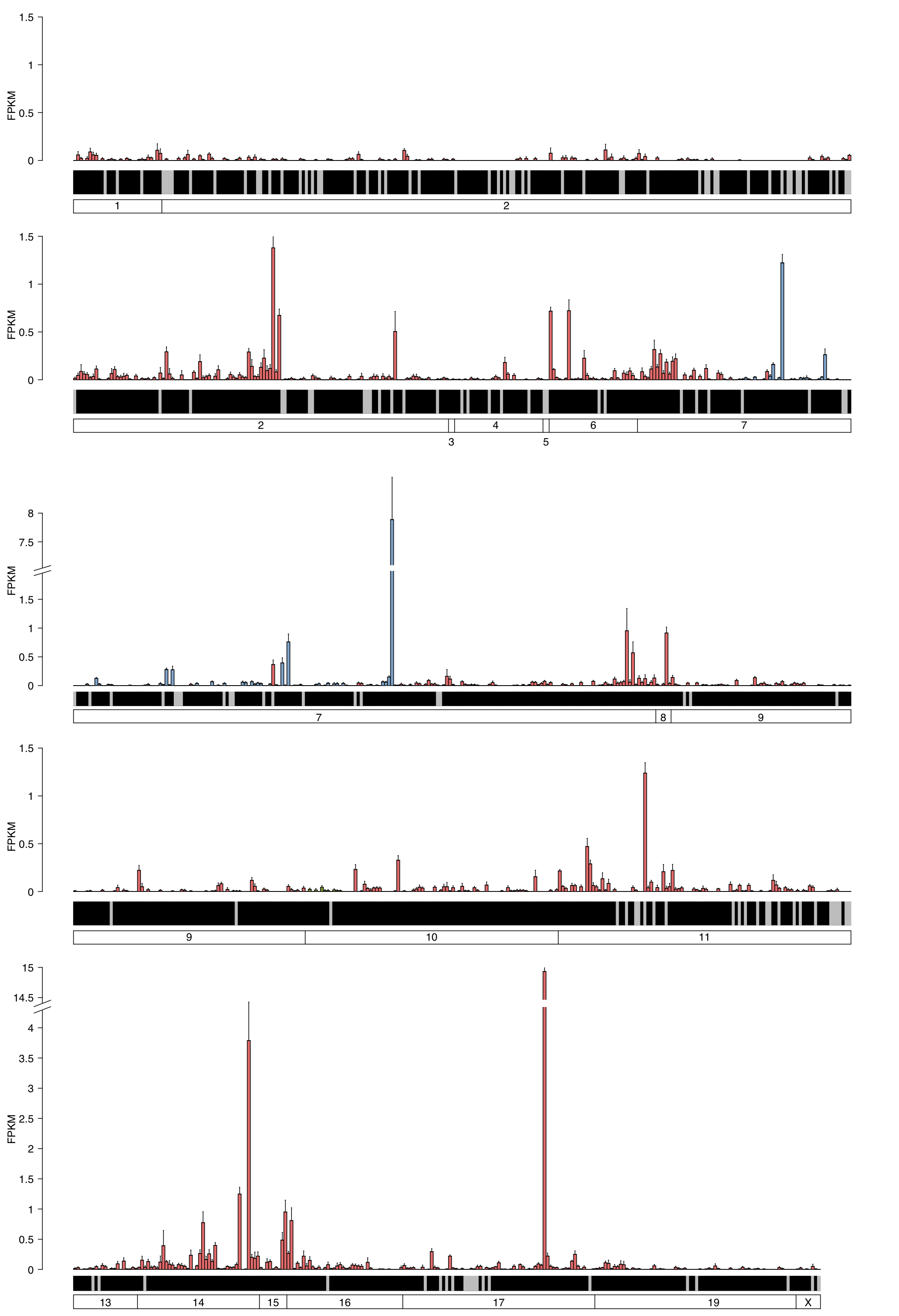

Supplement: Figure S5 — Expression of the complete OR repertoire in the VNO. The mean FPKM expression values are shown for all the OR genes and trace amine-associated receptor (TAAR) genes in the VNO; error bars represent the standard error over the mean from the six biological replicates. Genes are ordered by their chromosomal location and chromosomes are annotated in the boxes at the bottom. Class I OR genes are colored in blue, class II OR genes in red and TAAR genes in green. Below the plot, the black shading indicates the gene is annotated as a functional receptor, and grey is for pseudogenes. (TIFF) [file pgen.1004593.s005.tiff]

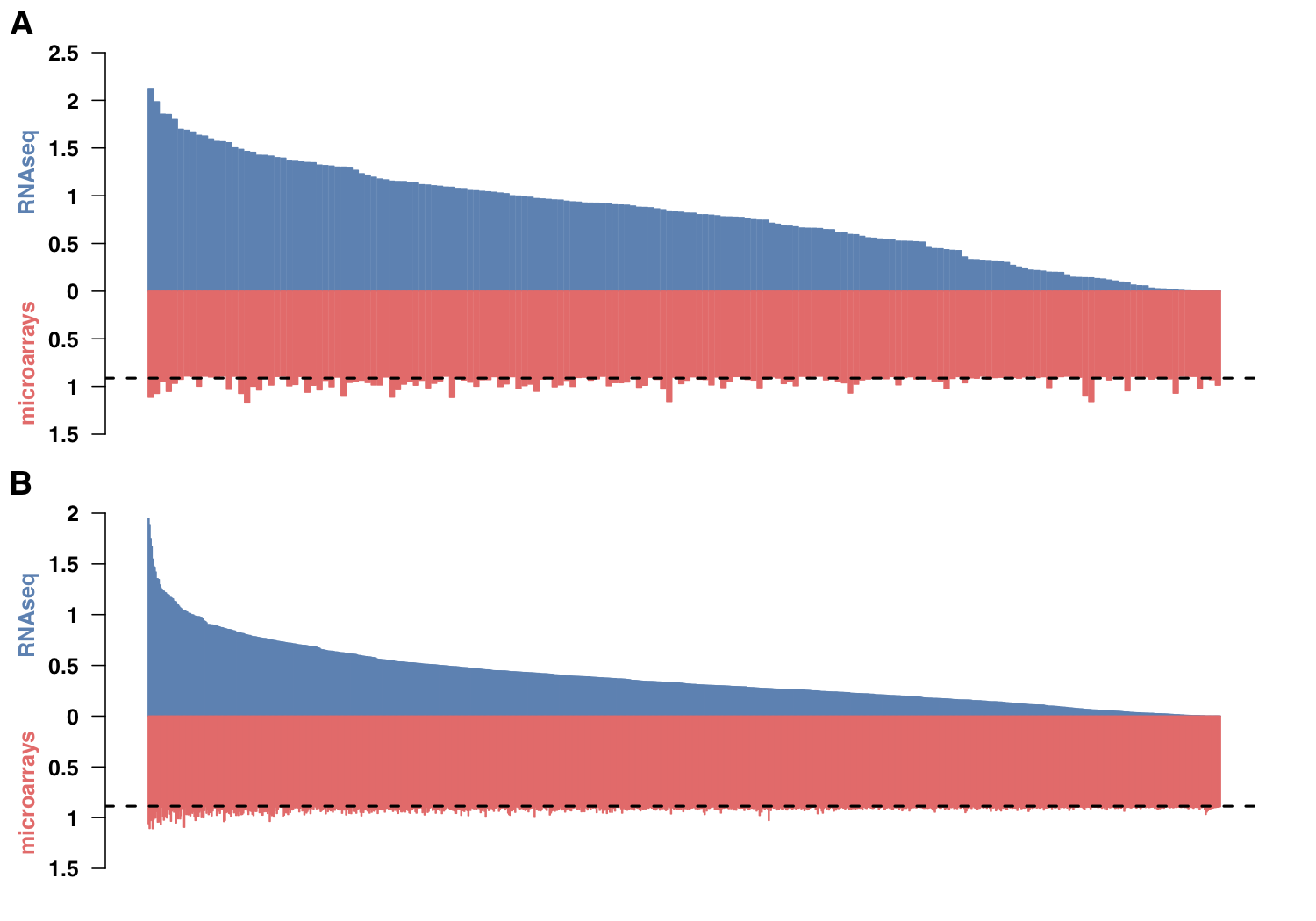

Supplement: Figure S6 — Expression of VR and OR genes in RNAseq compared with microarray expression data. Expression of the VR (A) and OR (B) genes that are present in both the microarray and the RNAseq data. The log10 FPKM+1 values from the RNAseq data are plotted in blue, and genes are ordered by decreasing expression level. In red is the log10 normalized intensity values from Illumina expression microarrays for the corresponding genes. The dotted black line represents the background intensity level from the microarray. Gene expression values lower than this threshold cannot be distinguished from noise. (TIFF) [file pgen.1004593.s006.tiff]

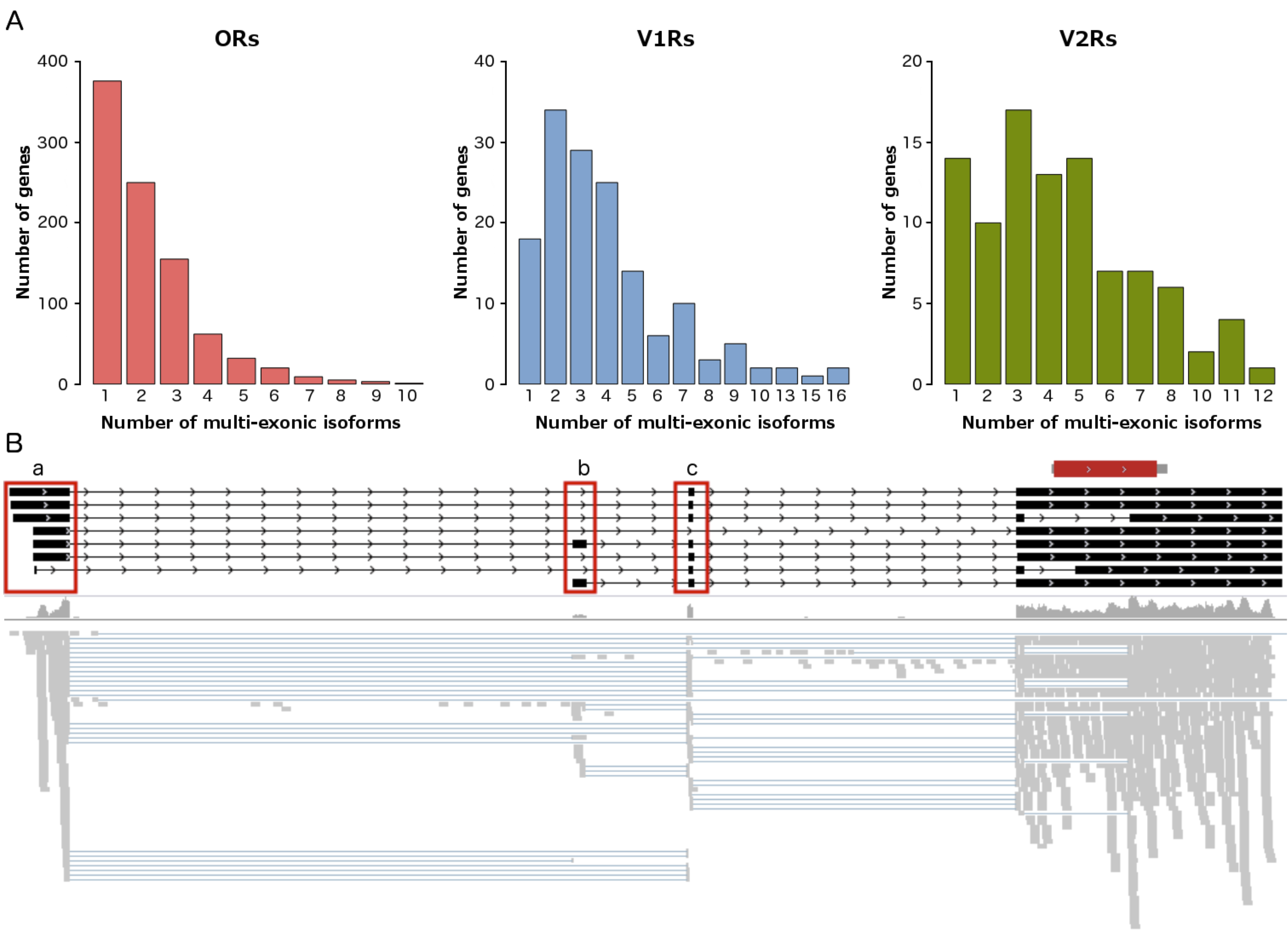

Supplement: Figure S7 — Alternative splicing in VR and OR genes. (A) Histograms representing the number of receptor genes that show different number of multi-exonic isoforms for OR, V1R and V2R genes. (B) Most genes with a high number of isoforms arise from multiple combinations of slight variations in the (a) transcription start site, (b) the differential inclusion of specific exons and (c) the difference in splice sites, which produces exons that differ in length by a few nucleotides. Data shown for Olfr1420 as an example; in red is the Ensembl gene model, with the UTR regions in grey, and in black the models produced by Cufflinks. Below are the sequencing reads in grey, and blue lines join reads that span exon junctions. (TIFF) [file pgen.1004593.s007.tiff]

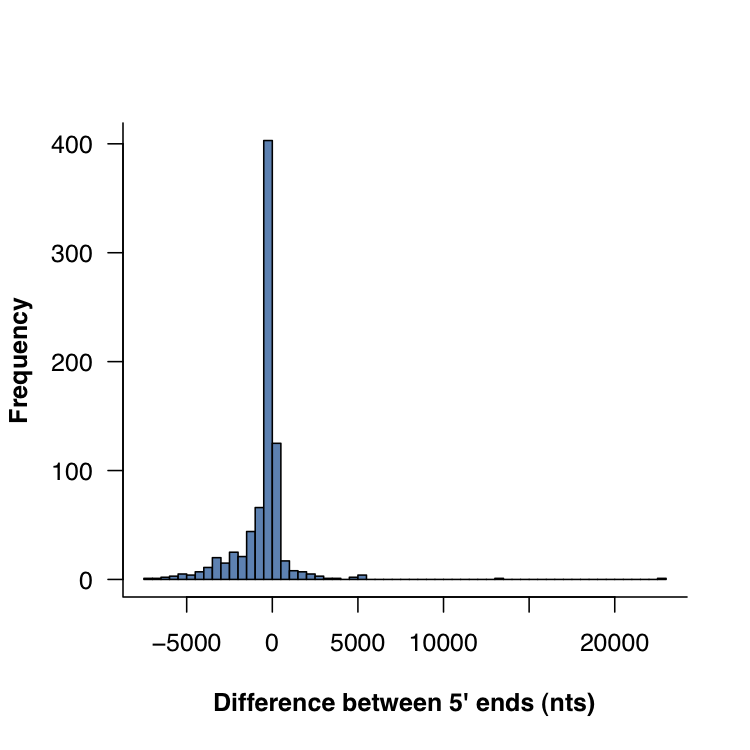

Supplement: Figure S8 — Comparison of the 5′ end of the OR genes as inferred by Cufflinks or as reported by Clowney et al. (2011). The difference in nucleotides between the two ends was calculated; a negative value indicates the 5′ end reported by Clowney et al. is upstream of the one reported here, by Cufflinks [33]. (TIFF) [file pgen.1004593.s008.tiff]

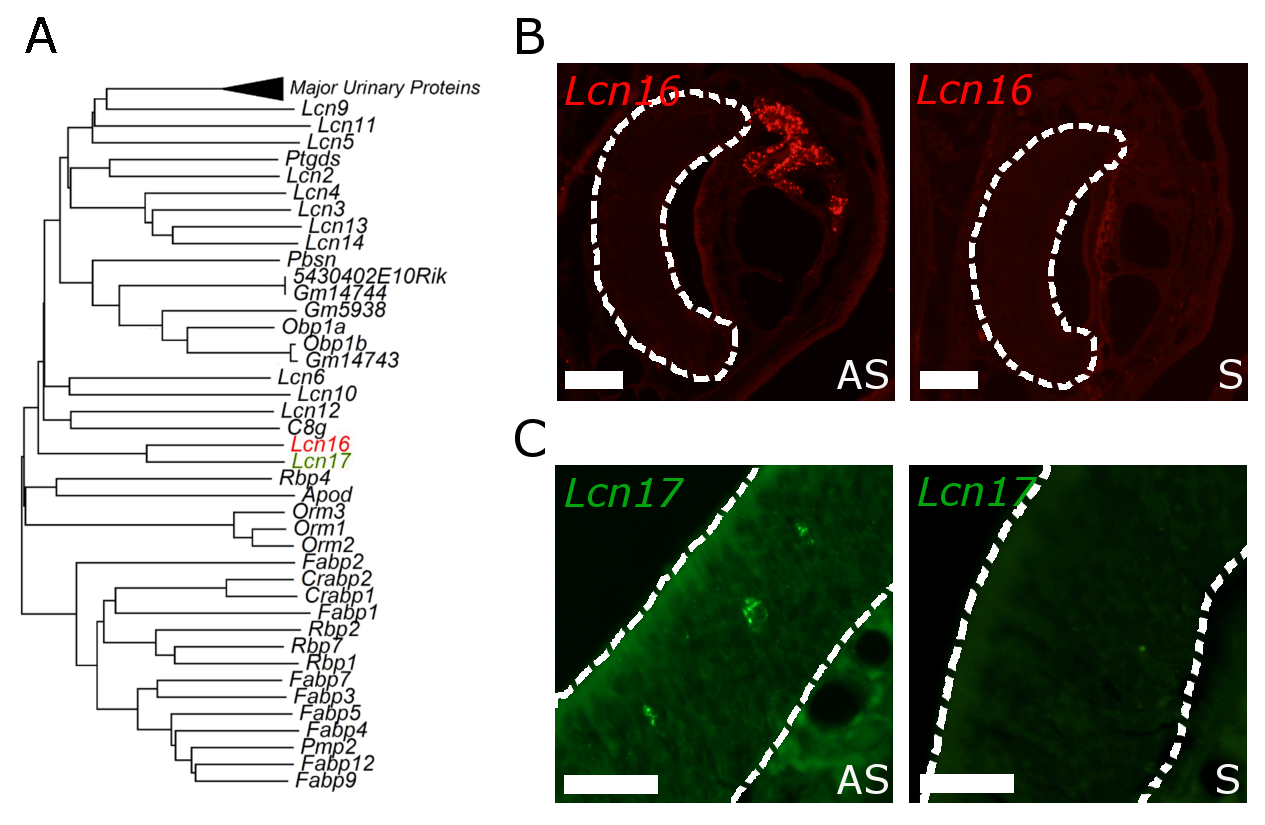

Supplement: Figure S9 — Novel lipocalins are expressed in olfactory tissues. (A) Phylogenetic reconstruction of the novel genes with other members of the mouse lipocalin gene family. In situ hybridization with an antisense probe (AS, left) reveals (B) Lcn16 is expressed in glandular tissue of the VNO, but not within the sensory epithelium (dashed line) and (C) Lcn17 is expressed within the main olfactory epithelium of the OM (dashed line). No signals are detected using the corresponding sense probes (S, right). Scale bars: (B) 100 µm, (C) 50 µm. (TIF) [file pgen.1004593.s009.tif]
