# Supplementary material for: The Olfactory Transcriptomes of Mice
Source: PLoS Genet. 2014 Sep 4;10(9):e1004593. doi: 10.1371/journal.pgen.1004593 (PMC4154679; doi:10.1371/journal.pgen.1004593)
Supplement: Table S1 — TaqMan probes used in qRT-PCR assays. The IDs of the specific assays used are provided. (DOCX) [file pgen.1004593.s010.docx]

**Table S1. TaqMan probes used in qRT-PCR assays.**

| **Ensembl ID** | **Gene name** | **TaqMan probe ID** |
| --- | --- | --- |
| **ENSMUSG00000029580** | ***Actb*** | **Mm00607939_s1** |
| **ENSMUSG00000060802** | ***B2m*** | **Mm00437762_m1** |
| **ENSMUSG00000027483** | ***Bpifa1*** | **Mm00465064_m1** |
| **ENSMUSG00000001175** | ***Calm1*** | **Mm01336281_g1** |
| **ENSMUSG00000031628** | ***Casp3*** | **Mm01195085_m1** |
| **ENSMUSG00000056025** | ***Clca1*** | **Mm00777368_m1** |
| **ENSMUSG00000005864** | ***Cnga2*** | **Mm01281967_m1** |
| **ENSMUSG00000047517** | ***Dmbt1*** | **Mm00455996_m1** |
| **ENSMUSG00000005980** | ***Dnase1*** | **Mm01342387_g1** |
| **ENSMUSG00000037742** | ***Eef1a1*** | **Mm01973893_g1** |
| **ENSMUSG00000000983** | ***Expi*** | **Mm00433159_m1** |
| **ENSMUSG00000032562** | ***Gnai2*** | **Mm01232052_g1** |
| **ENSMUSG00000024524** | ***Gnal*** | **Mm01258317_m1** |
| **ENSMUSG00000031748** | ***Gnao1*** | **Mm00494677_m1** |
| **ENSMUSG00000020890** | ***Gucy2e*** | **Mm00433868_m1** |
| **ENSMUSG00000036594** | ***H2-Aa*** | **Mm00439211_m1** |
| **ENSMUSG00000032496** | ***Ltf*** | **Mm00434787_m1** |
| **ENSMUSG00000006476** | ***Nelf*** | **Mm00480348_g1** |
| **ENSMUSG00000089751** | ***Olfr1213*** | **Mm00654667_s1** |
| **ENSMUSG00000045202** | ***Olfr123*** | **Mm00730230_s1** |
| **ENSMUSG00000044985** | ***Olfr124*** | **Mm0052996_s1** |
| **ENSMUSG00000051313** | ***Olfr1262*** | **Mm00654960_s1** |
| **ENSMUSG00000034583** | ***Olfr1347*** | **Mm01700905_s1** |
| **ENSMUSG00000059887** | ***Olfr1507*** | **Mm00451539_s1** |
| **ENSMUSG00000035626** | ***Olfr1509*** | **Mm00451556_s1** |
| **ENSMUSG00000059610** | ***Olfr222*** | **Mm02391416_s1** |
| **ENSMUSG00000043948** | ***Olfr691*** | **Mm00529987_s1** |
| **ENSMUSG00000073906** | ***Olfr692*** | **Mm00526312_s1** |
| **ENSMUSG00000074006** | ***Omp*** | **Mm00448081_s1** |
| **ENSMUSG00000054640** | ***Slc8a1*** | **Mm01232254_m1** |
| **ENSMUSG00000039954** | ***Stk32a*** | **Mm01258055_m1** |
| **ENSMUSG00000070425** | ***Trpc2*** | **Mm00441984_m1** |
| **ENSMUSG00000041596** | ***Vmn1r90*** | **Mm01180791_m1** |
| **ENSMUSG00000027824** | ***Vmn2r1*** | **Mm00498222_m1** |
| **ENSMUSG00000095730** | ***Vmn2r29*** | **Mm02525417_g1** |
| **ENSMUSG00000091572** | ***Vmn2r3*** | **Mm01294907_m1** |
| **ENSMUSG00000086503** | ***Xist*** | **Mm01232884_m1** |
| **ENSMUSG00000041333** | ***Mup4*** | **Mm00657760_gH** |
| **ENSMUSG00000058523** | ***Mup5*** | **Mm01702733_g1** |
| **ENSMUSG00000067684** | ***Obp1a*** | **Mm00500903_m1** |
| **ENSMUSG00000044121** | ***5430402E10Rik*** | **Mm02745943_g1** |
