# Supplementary material for: The Olfactory Transcriptomes of Mice
Source: PLoS Genet. 2014 Sep 4;10(9):e1004593. doi: 10.1371/journal.pgen.1004593 (PMC4154679; doi:10.1371/journal.pgen.1004593)
Supplement: Table S2 — VR genes are not properly annotated in Ensembl. Ensembl genes that match VR cDNA sequences but are annotated as ‘novel genes’. Those genes that match a sequence with 100% identity were included in our analyses and their name was changed to that of the cDNA sequence they matched (third column). (DOCX) [file pgen.1004593.s011.docx]

**Table S2. VRs are not all correctly annotated in databases.**

Ensembl genes that match VR cDNA sequences but are annotated as ‘novel genes’. Those genes that match a sequence with 100% identity were included in our analyses and their name was changed to that of the cDNA sequence they matched (third column).

| **Ensembl ID** | **Ensembl gene name** | **VR cDNA with 100% identity** |
| --- | --- | --- |
| **ENSMUSG00000095451** | ***AC107868.1*** | ***Vmn1r227*** |
| **ENSMUSG00000096294** | ***Gm10302*** | ***Vmn2r47*** |
| **ENSMUSG00000096871** | ***Gm10665*** | ***Vmn1r102*** |
| **ENSMUSG00000096348** | ***Gm10666*** | ***Vmn1r141.Vmn1r93*** |
| **ENSMUSG00000094762** | ***Gm10670*** | ***Vmn1r150*** |
| **ENSMUSG00000087688** | ***Gm11300*** | ***Vmn1r203*** |
| **ENSMUSG00000087643** | ***Gm11314*** | ***Vmn1r208*** |
| **ENSMUSG00000082316** | ***Gm11329*** | ***Vmn1r221*** |
| **ENSMUSG00000096152** | ***Gm16442*** | ***Vmn1r140*** |
| **ENSMUSG00000095745** | ***Gm4133*** | ***Vmn1r146*** |
| **ENSMUSG00000095837** | ***Gm4141*** | ***Vmn1r106*** |
| **ENSMUSG00000093941** | ***Gm4172*** | ***Vmn1r131*** |
| **ENSMUSG00000096513** | ***Gm4175*** | ***Vmn1r133*** |
| **ENSMUSG00000096760** | ***Gm4177*** | ***Vmn1r134*** |
| **ENSMUSG00000095163** | ***Gm4179*** | ***Vmn1r138*** |
| **ENSMUSG00000093871** | ***Gm4187*** | ***Vmn1r98*** |
| **ENSMUSG00000095984** | ***Gm4201*** | ***Vmn1r154*** |
| **ENSMUSG00000092297** | ***Gm4214*** | ***Vmn1r161*** |
| **ENSMUSG00000094532** | ***Gm4216*** | ***Vmn1r162*** |
| **ENSMUSG00000096073** | ***Gm4220*** | ***Vmn1r166*** |
| **ENSMUSG00000094757** | ***Gm4498*** | ***Vmn1r145*** |
| **ENSMUSG00000095191** | ***Gm5725*** | ***Vmn1r136*** |
| **ENSMUSG00000096761** | ***Gm5726*** | ***Vmn1r105*** |
| **ENSMUSG00000095806** | ***Gm5728*** | ***Vmn1r147*** |
| **ENSMUSG00000094298** | ***Gm6164*** | ***Vmn1r144*** |
| **ENSMUSG00000094149** | ***Gm8453*** | ***Vmn1r97*** |
| **ENSMUSG00000094981** | ***Gm8653*** | ***Vmn1r96*** |
| **ENSMUSG00000093917** | ***Gm8660*** | ***Vmn1r99*** |
| **ENSMUSG00000094748** | ***Gm8677*** | ***Vmn1r153*** |
| **ENSMUSG00000095081** | ***Gm8693*** | ***Vmn1r108.Vmn1r156*** |
| **ENSMUSG00000096601** | ***Gm8720*** | ***Vmn1r164*** |
| **ENSMUSG00000091528** | ***Gm9268*** | ***Vmn2r64*** |
| **ENSMUSG00000096304** | ***RP23-331M13.5*** | ***Vmn1r92*** |
| **ENSMUSG00000092456** | ***V1rd19*** | ***Vmn1r182*** |
| **ENSMUSG00000069295** | ***V1rh19*** | ***Vmn1r196*** |
