# Supplementary material for: The Olfactory Transcriptomes of Mice
Source: PLoS Genet. 2014 Sep 4;10(9):e1004593. doi: 10.1371/journal.pgen.1004593 (PMC4154679; doi:10.1371/journal.pgen.1004593)

Dataset S8

OR and VR transcripts that have introns within the coding sequence.

'Not expressed' means there are not enough sequencing reads to conclude whether the intron is supported or not.

geneID	transcriptID	geneName	reconstructed transcriptID	intron in coding sequence	comments
ENSMUSG00000068810	ENSMUST00000090702	Olfr1187-ps1	--	not expressed	
ENSMUSG00000068810	ENSMUST00000177576	Olfr1187-ps1	--	not expressed	
ENSMUSG00000081724	ENSMUST00000174675	Olfr129	CUFFORT_0705; CUFFORT_0706	supported	The transcript annotated in Ensembl is truncated and produces a 70 aa protein; The reconstructed transcripts have the same intron but produce full length ORFs (320 aa).
ENSMUSG00000063240	ENSMUST00000173610	Olfr133	CUFFORT_0648	supported	The transcript annotated in Ensembl is truncated and produces a 109 aa protein; The reconstructed transcripts have the same intron but produce a full length ORF (327 aa).
ENSMUSG00000073769	ENSMUST00000106360	Olfr1331	CUFFORT_1394	supported	
ENSMUSG00000073770	ENSMUST00000106361	Olfr1333	CUFFORT_1413	supported	
ENSMUSG00000093877	ENSMUST00000086319	Olfr1348	CUFFORT_2049; CUFFORT_2050	supported	
ENSMUSG00000063583	ENSMUST00000079790	Olfr1410	CUFFORT_0008	**not supported**	
ENSMUSG00000066896	ENSMUST00000086473	Olfr18	CUFFORT_1948	supported	
ENSMUSG00000090129	ENSMUST00000170618	Olfr287	CUFFORT_2020	supported	
	ENSMUST00000155603				
	ENSMUST00000141911				
ENSMUSG00000075427	ENSMUST00000165379	Olfr288	--	supported	

ENSMUSG00000061549	ENSMUST00000073222	Olfr301	CUFFORT_1480	supported	
ENSMUSG00000063881	ENSMUST00000078952	Olfr376	CUFFORT_0282	supported	
ENSMUSG00000059623	ENSMUST00000071725	Olfr39	--	supported	
ENSMUSG00000073962	ENSMUST00000098213	Olfr576	--	**not supported**	
ENSMUSG00000041885	ENSMUST00000042676	Olfr680-ps1	--	not expressed	
ENSMUSG00000059768	ENSMUST00000071242	Olfr682-ps1	--	not expressed	
ENSMUSG00000066914	ENSMUST00000086496	Olfr831-ps1	--	not expressed	
ENSMUSG00000058491	ENSMUST00000075717	Olfr869	--	supported	
ENSMUSG00000066897	ENSMUST00000086474	Olfr872	CUFFORT_1855	supported	
ENSMUSG00000096356	ENSMUST00000072290	Olfr889	CUFFORT_1876	**not supported**	
ENSMUSG00000050813	--	Olfr332	CUFFORT_0312	lowly supported	This transcript hasn't been reported in Ensembl. The intron leads to a deletion of 28 aa; the product might or might not be a functional receptor
ENSMUSG00000079064	ENSMUST00000110449	Vmn1r-ps123	--	not expressed	
ENSMUSG00000091638	ENSMUST00000168794	Vmn1r113	--	not expressed	
ENSMUSG00000074311	ENSMUST00000173668	Vmn1r139	--	supported	
ENSMUSG00000091662<br>ENSMUSG00000091662	ENSMUST00000176284<br>ENSMUST00000176707	Vmn1r69	CUFFVRT_0890, CUFFVRT_0892, CUFFVRT_0893	supported	The transcripts annotated in Ensembl are truncated and produce a 57 aa protein; The reconstructed transcripts have the same intron but produce full length ORFs (338 aa).

The IGV screenshots with the mapped reads to the gene models are below. The Ensembl gene models are in red and additional transcripts reconstructed by Cufflinks are in black. The non coding regions are in grey for all. Sequencing fragments are drawn in grey below, and the blue lines join reads that span exon junction. For the genes where the intron is not supported all six sequenced samples are shown. For all others, a representative sample is presented.

Olfr129 – Supported

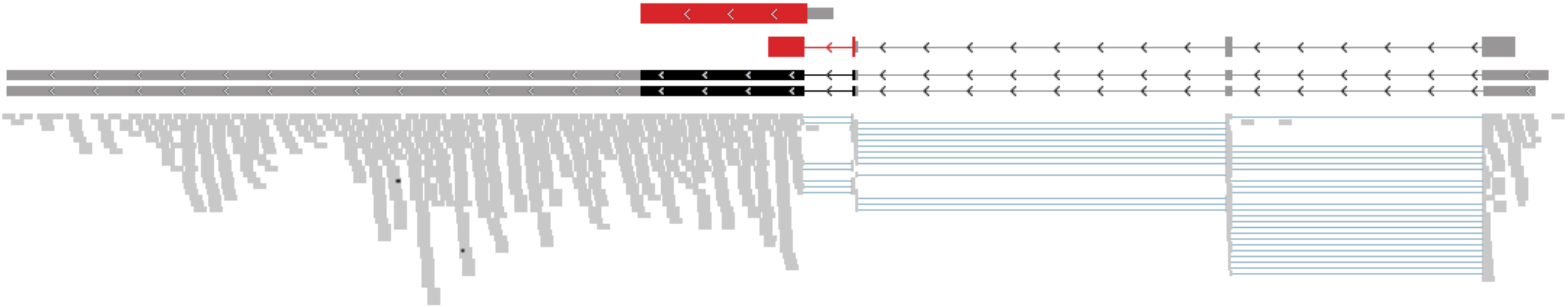

Olfr133 – Supported

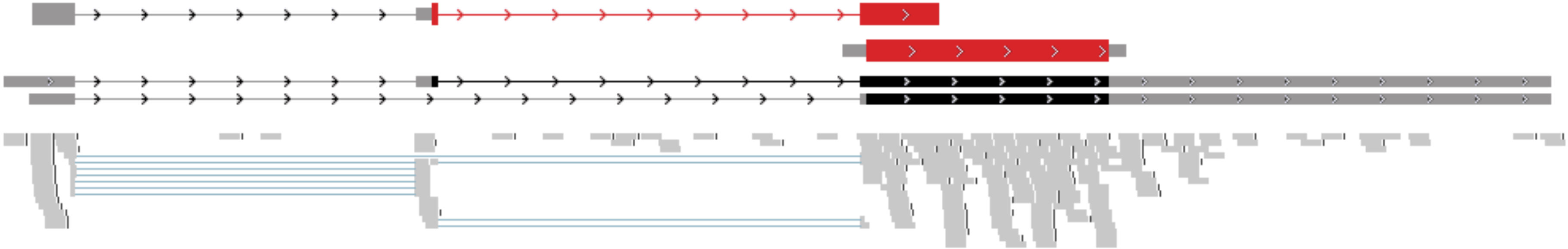

Olfr1331 – Supported

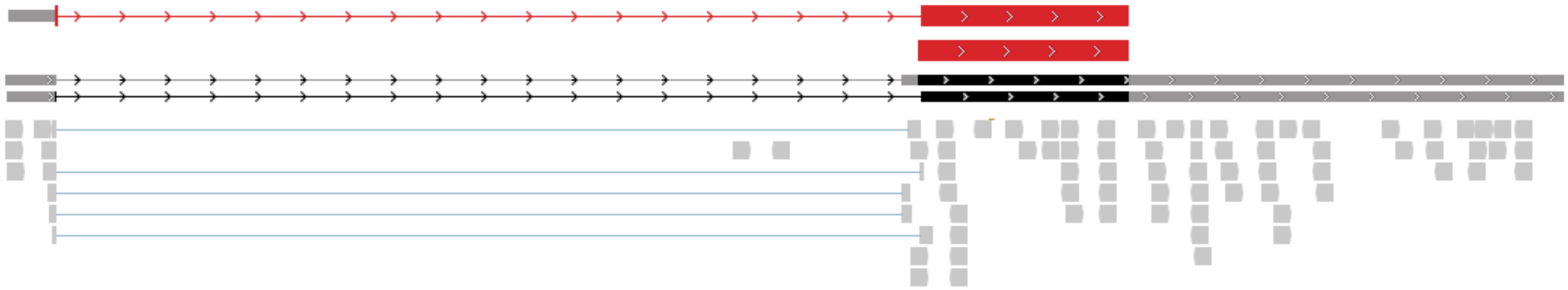

Olfr1333 – Supported

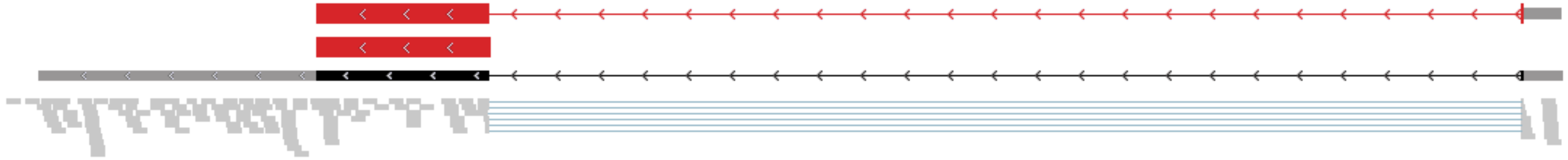

Olfr18 – Supported

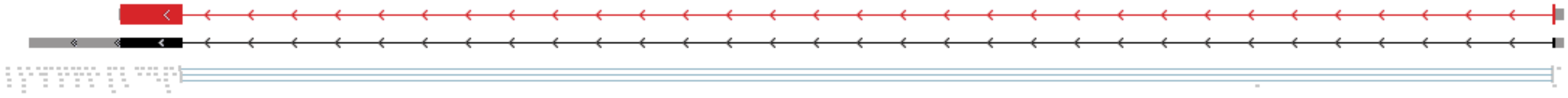

Olfr1348 – Supported

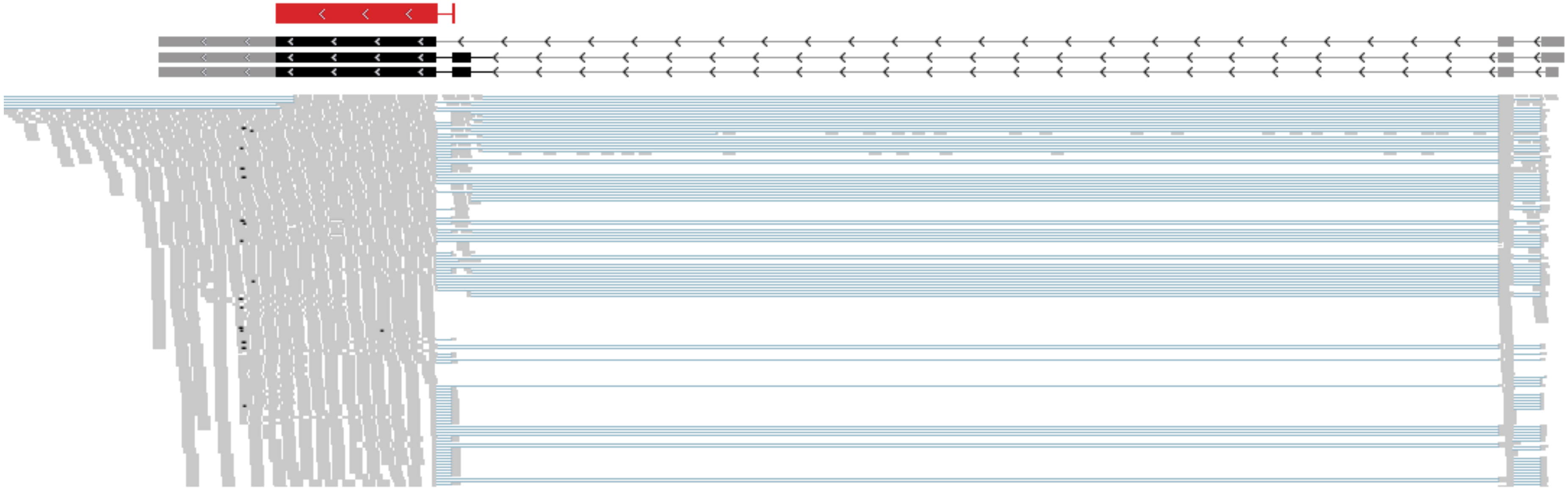

Olfr1410 – Not supported

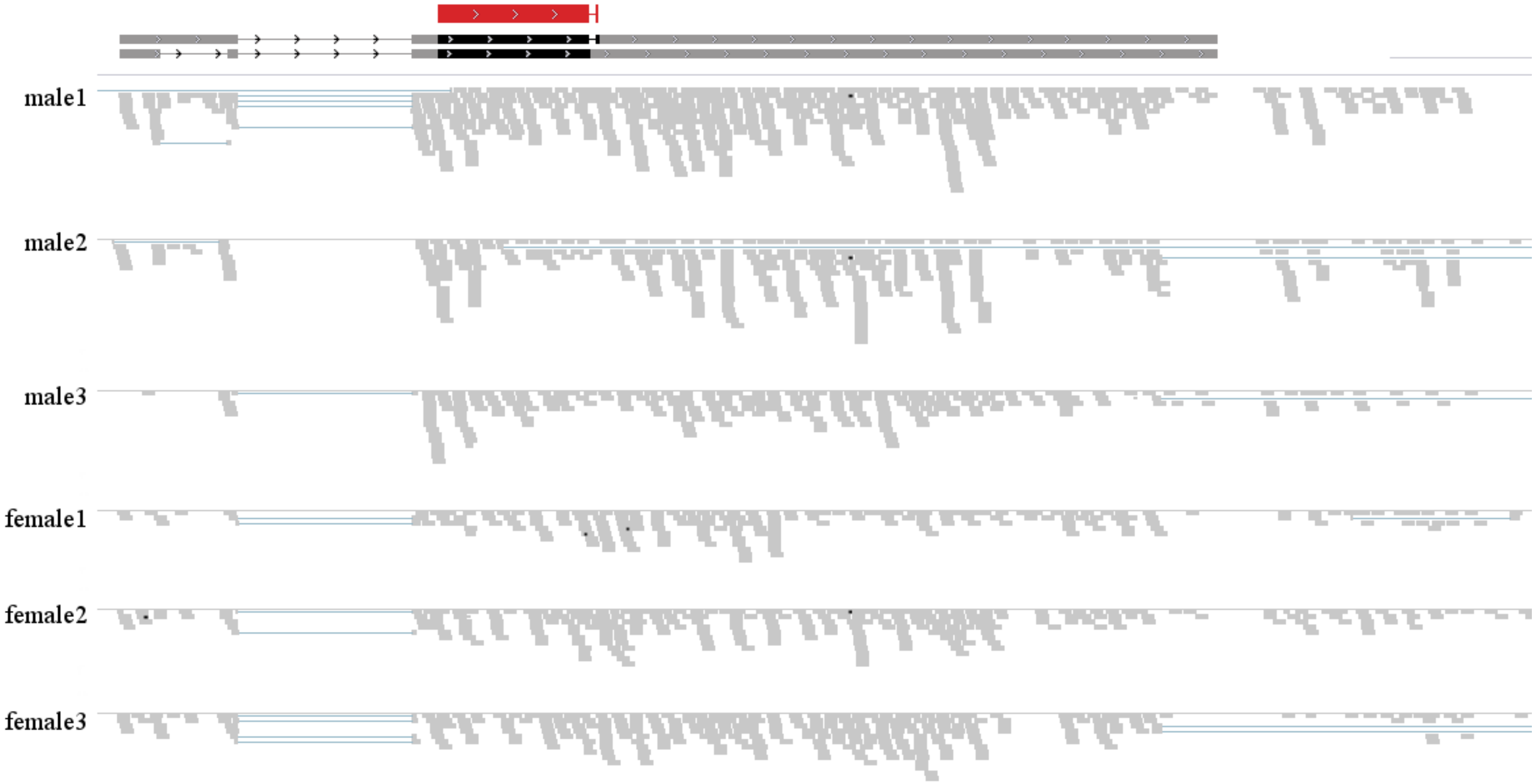

Olfr287 – Supported

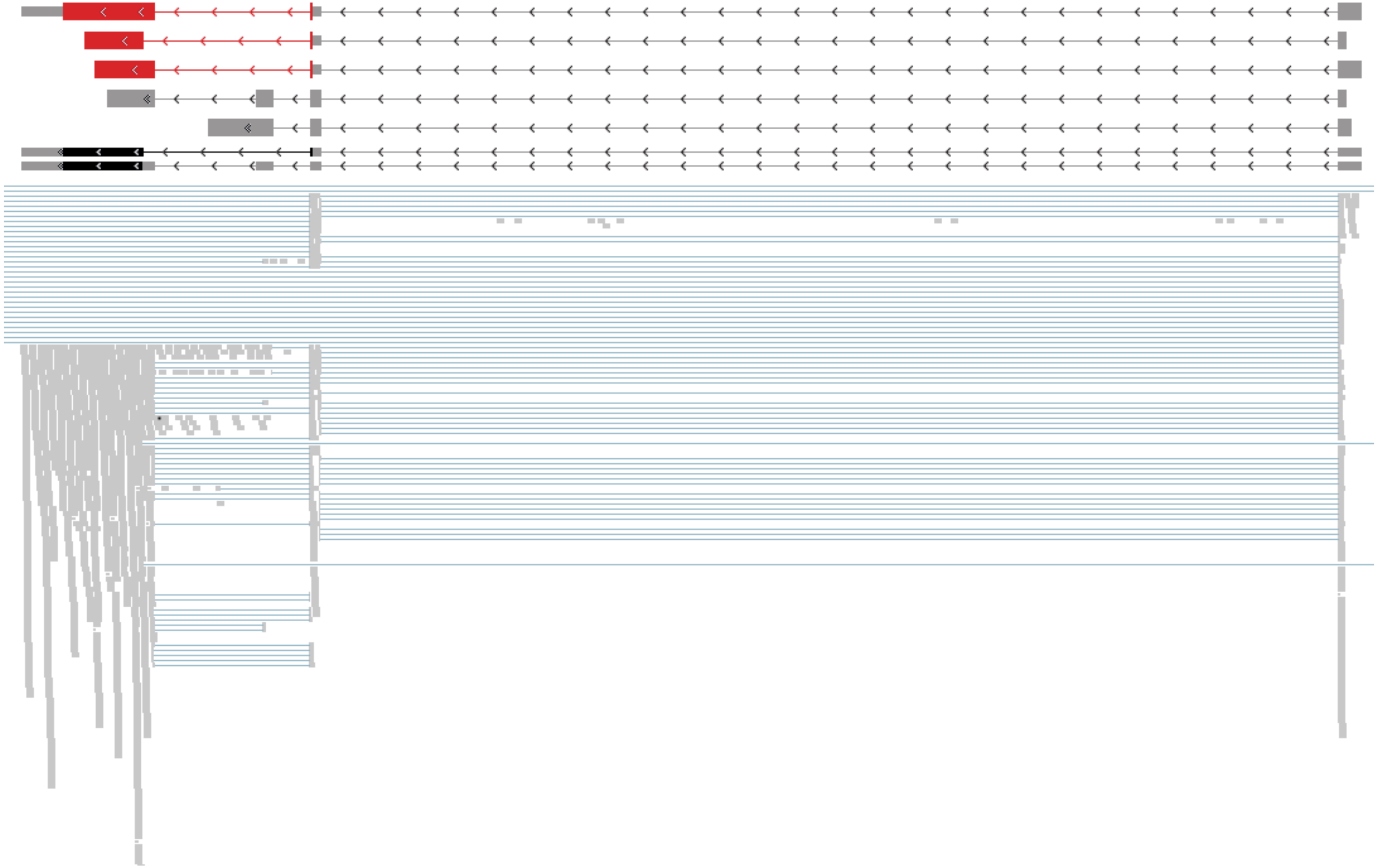

Olfr288 – Supported

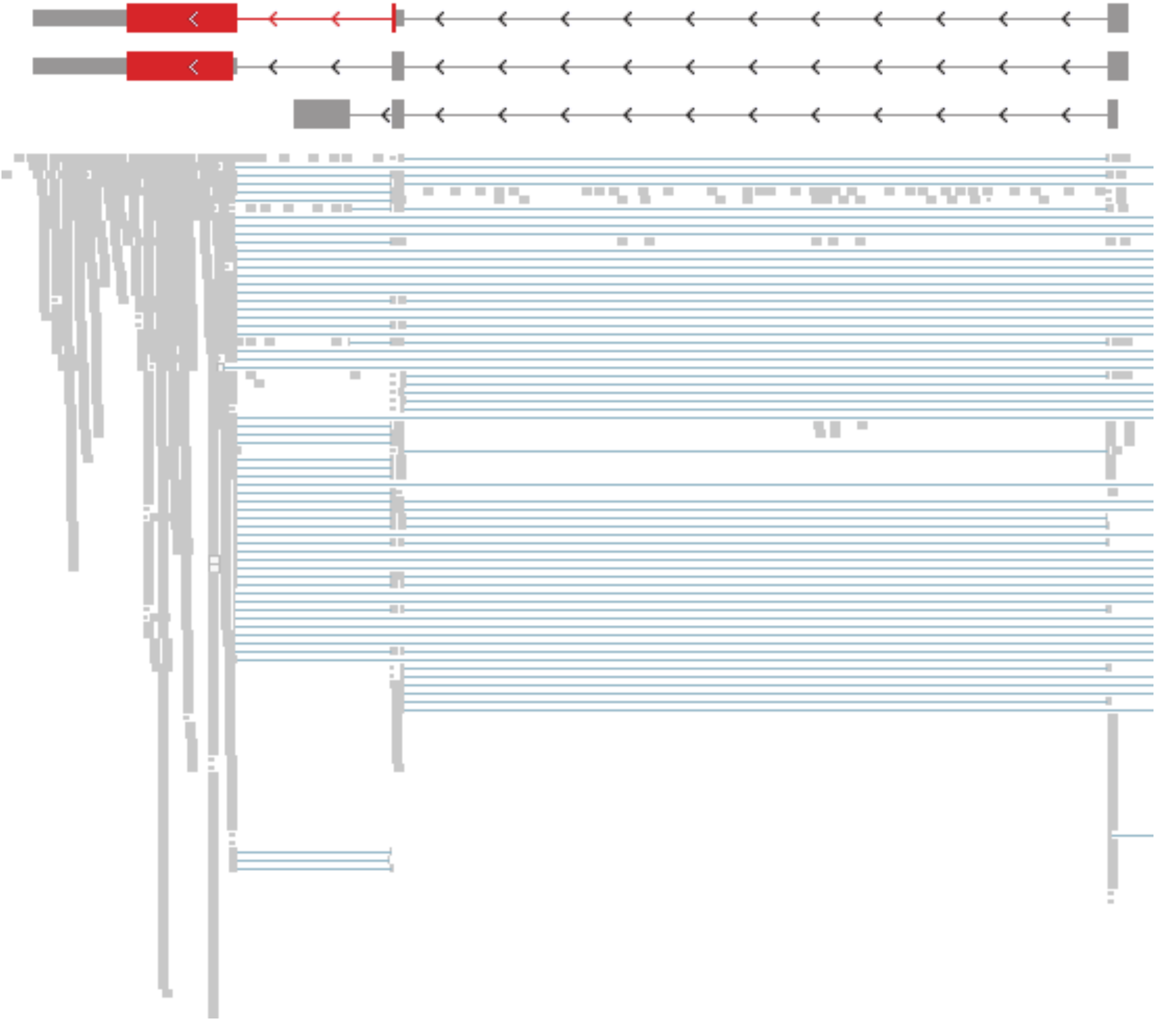

Olfr301 – Supported

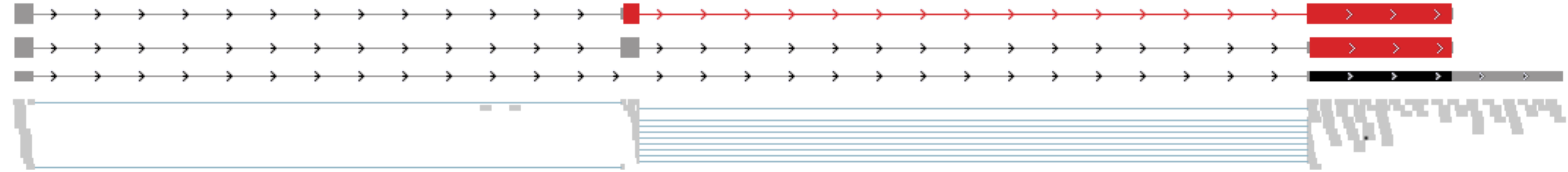

Olfr376 – Supported

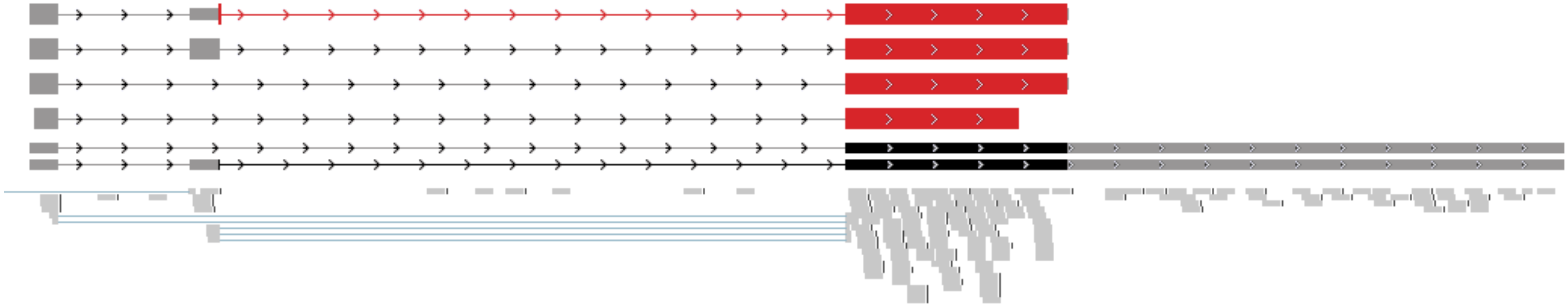

Olfr39 – Supported

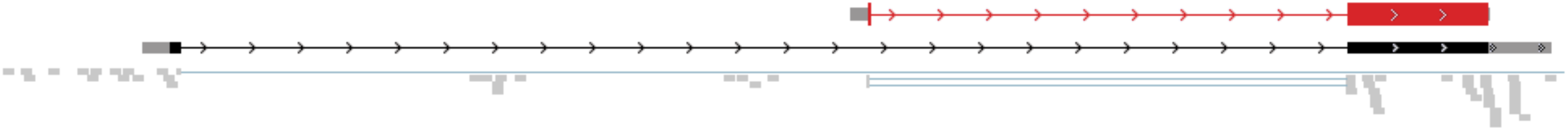

Olfr576 – Not supported

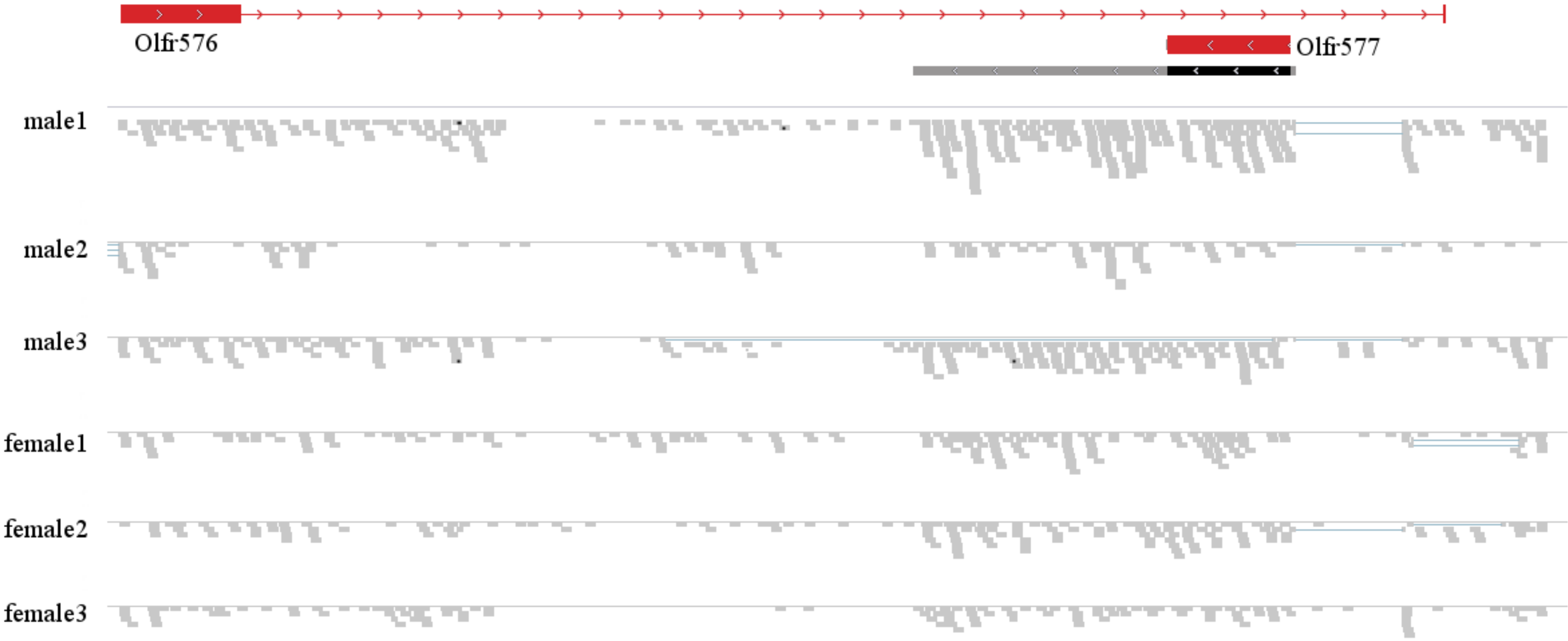

Olfr869 – Supported

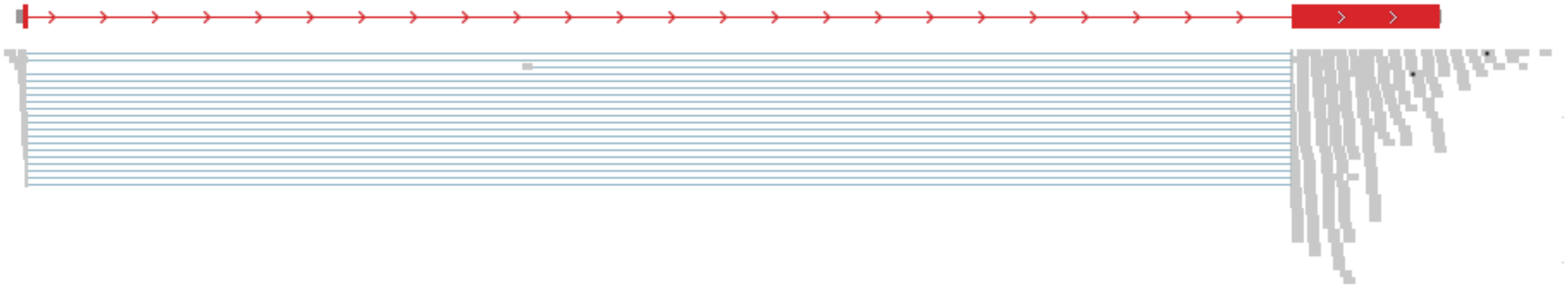

Olfr872 – Supported

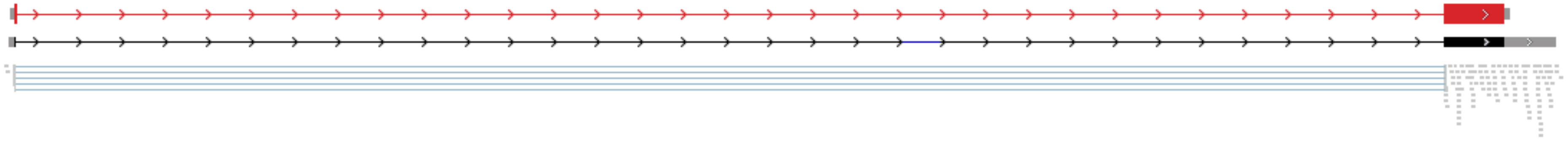

Olfr889 – Not supported

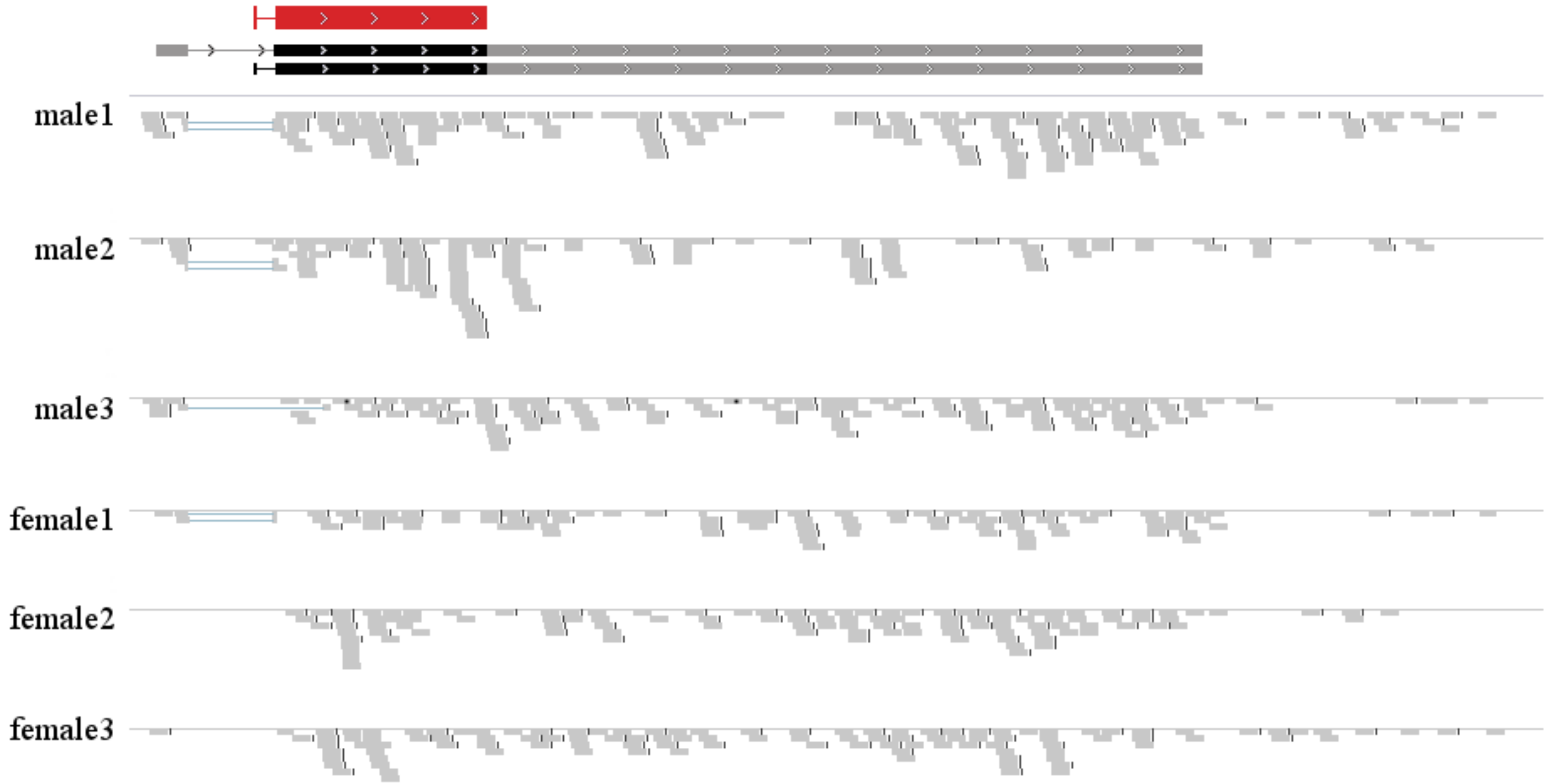

Olfr332 – Lowly supported

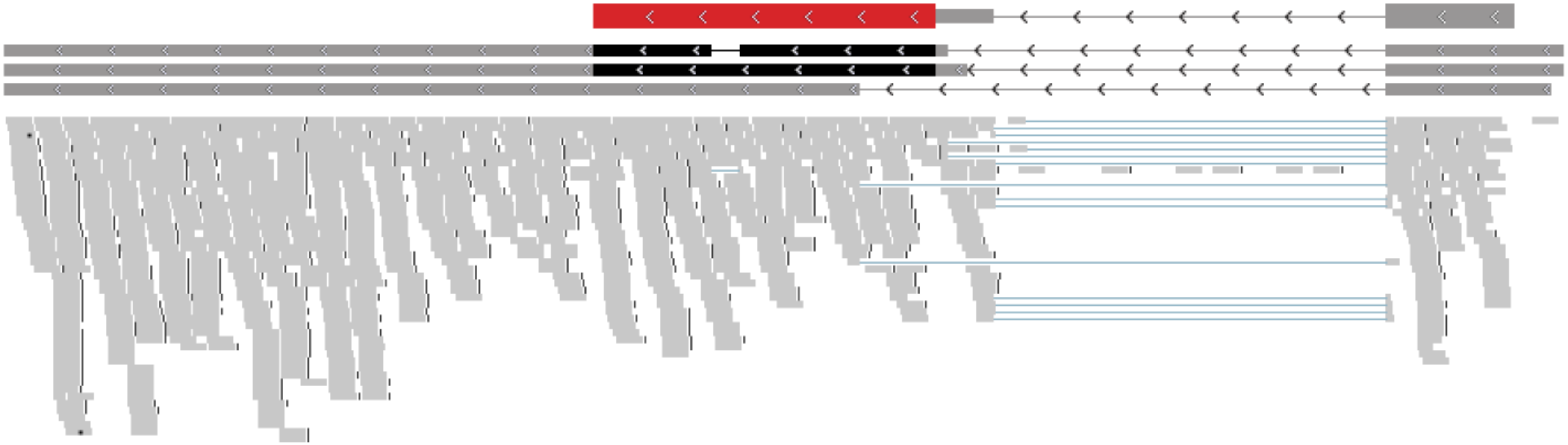

Vmn1r69 - Supported

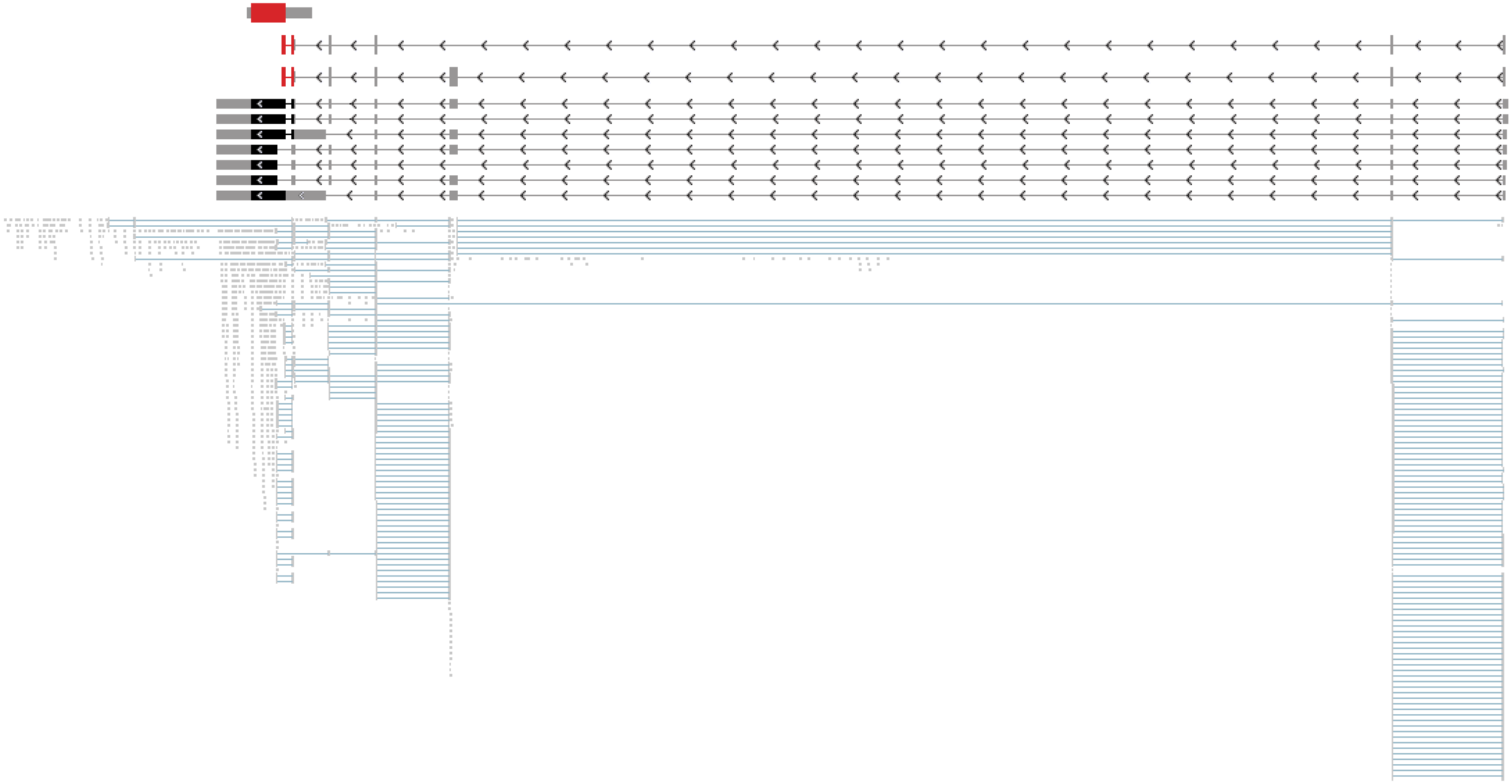

Vmn1r139 – Supported (only the relevant part of the gene model is shown, as the whole transcript is > 28Kb long)

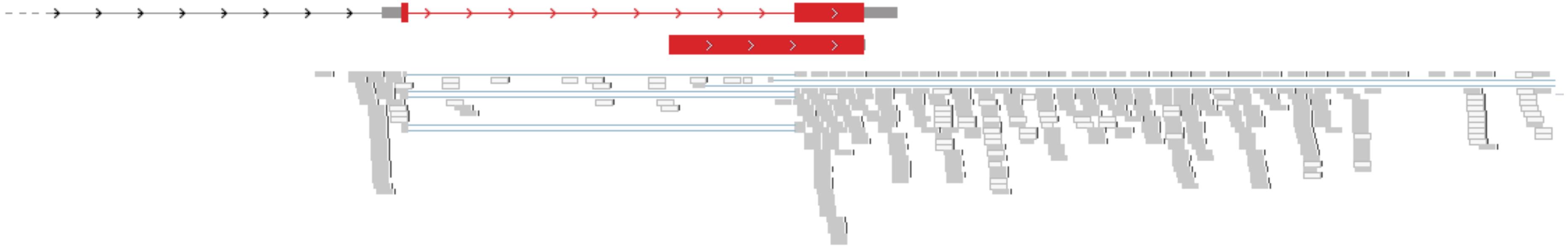

Supplement: Dataset S8 — List of VR and OR genes that have introns within the annotated coding sequence. The genes and their identifiers are listed, and screenshots from IGV with the visualization of the reads supporting the introns are presented. (PDF) [file pgen.1004593.s019.pdf]
