# Supplementary material for: The Olfactory Transcriptomes of Mice
Source: PLoS Genet. 2014 Sep 4;10(9):e1004593. doi: 10.1371/journal.pgen.1004593 (PMC4154679; doi:10.1371/journal.pgen.1004593)

Dataset S9

We compared the 5’ end of the OR gene models reconstructed by Cufflinks with the 5’ ends proposed by Plessy et al. (2012) obtained through nanoCAGE. We inspected the sequencing data for the 25 genes with the largest differences in the proposed 5’ end. The discrepancy can be explained bay one of four scenarios:

Scenario	Reason for discrepancy	# of cases
1	reads support Cufflinks	10
2	reads support Cufflinks<br>nanoCAGE 5' overlaps 3' of adjacent OR	12
3	5' end of a different gene	2
4	reads support nanoCAGE	1
		25

We show a representative example of each scenario below. In red are the Ensembl gene models. In black is the reconstructed gene model by Cufflinks, if any. The non-coding regions are shown in grey. In blue is the data provided by Plessy et al. (2012). Arrows indicate whether the gene is in the forward or reverse strand. The sequencing reads are drawn below in grey, and blue lines join fragments that span exon junctions.

Scenario 1 – In these cases the sequencing data supports the reconstruction done by Cufflinks and there are no reads to support the proposed 5’ end in the nanoCAGE data.

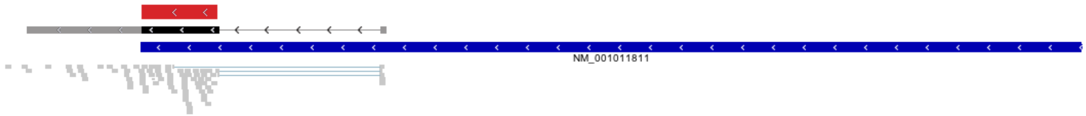

Scenario 2 – Similar to scenario 1, but the proposed 5’ end by nanoCAGE overlaps the 3’ end of an adjacent OR gene as proposed by Cufflinks. Data shown from Plessy et al. is for *Olfr12*.

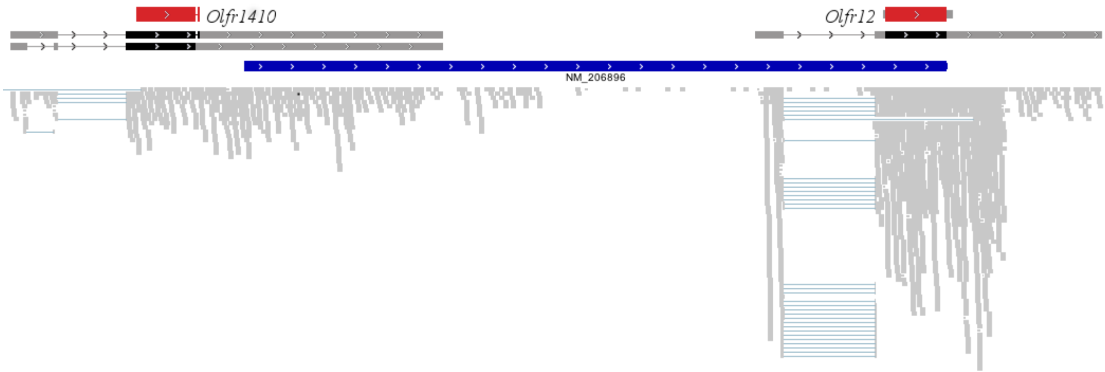

Scenario 3 – The 5' proposed by nanoCAGE actually corresponds to a different gene (that sometimes is not annotated in Ensembl) and not to the proposed OR.

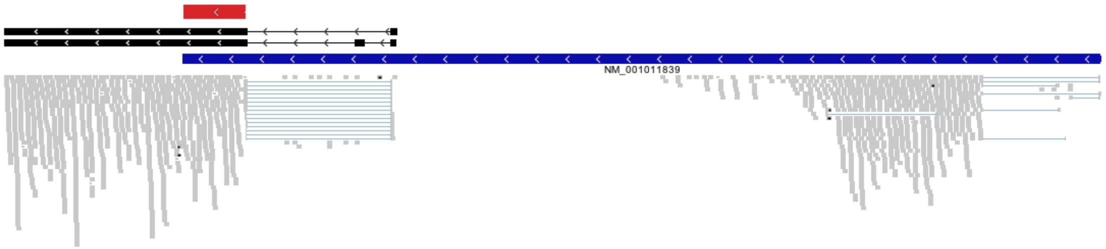

Scenario 4 – The sequencing reads support the end proposed by nanoCAGE and Cufflinks wasn't able to reconstruct the full-length model.

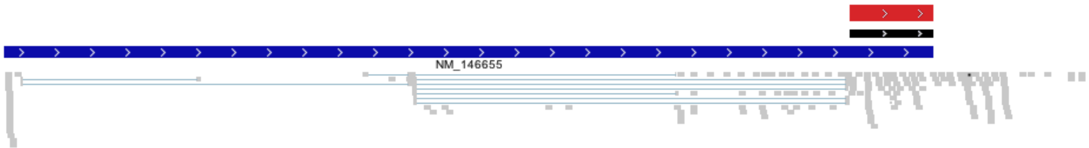

Supplement: Dataset S9 — Comparison to the Plessy et al. (2012) data. The OR genes with the largest difference in their predicted 5′ end by nanoCAGE or Cufflinks can be explained by four scenarios. These are detailed here and examples are provided. (PDF) [file pgen.1004593.s020.pdf]
